# Multiomic quantification of the *KRAS* mutation dosage improves the preoperative prediction of survival and recurrence in patients with pancreatic ductal adenocarcinoma

**DOI:** 10.1038/s12276-024-01382-0

**Published:** 2025-01-08

**Authors:** Won-Gun Yun, Daeun Kim, Youngmin Han, Wooil Kwon, Seong-Geun Lee, Jin-Young Jang, Daechan Park

**Affiliations:** 1 Department of Surgery and Cancer Research Institute, Seoul National University College of Medicine, Seoul, South Korea; 2 Department of Molecular Science and Technology, Ajou University, Suwon, South Korea; 3 Ajou Energy Science Research Center, Ajou University, Suwon, South Korea; 4 Advanced College of Bio-convergence Engineering, Ajou University, Suwon, South Korea

**Keywords:** Tumour biomarkers, Prognostic markers, Cancer genomics

## Abstract

Most cancer mutation profiling studies are laboratory-based and lack direct clinical application. For clinical use, it is necessary to focus on key genes and integrate them with relevant clinical variables. We aimed to evaluate the prognostic value of the dosage of the *KRAS* G12 mutation, a key pancreatic ductal adenocarcinoma (PDAC) variant and to investigate the biological mechanism of the prognosis associated with the dosage of the *KRAS* G12 mutation. In this retrospective cohort study, we analyzed 193 surgically treated patients with PDAC between 2009 and 2016. RNA, whole-exome, and *KRAS*-targeted sequencing data were used to estimate the dosage of the *KRAS* G12 mutant. Our prognostic scoring system included the mutation dosage from targeted sequencing ( > 0.195, 1 point), maximal tumor diameter at preoperative imaging ( > 20 mm, 1 point), and carbohydrate antigen 19-9 levels ( > 150 U/mL, 1 point). The *KRAS* mutation dosage exhibited comparable performance with clinical variables for survival prediction. High *KRAS* mutation dosages activated the cell cycle, leading to high mutation rates and poor prognosis. According to prognostic scoring systems that integrate mutation dosage with clinical factors, patients with 0 points had superior median overall survival of 97.0 months and 1-year, 3-year, and 5-year overall survival rates of 95.8%, 70.8%, and 66.4%, respectively. In contrast, patients with 3 points had worse median overall survival of only 16.0 months and 1-year, 3-year, and 5-year overall survival rates of 65.2%, 8.7%, and 8.7%, respectively. The incorporation of the *KRAS* G12 mutation dosage variable into prognostic scoring systems can improve clinical variable-based survival prediction, highlighting the feasibility of an integrated scoring system with clinical significance.

## KRAS mutant dosage enhances prediction of prognosis in pancreatic cancer

Pancreatic ductal adenocarcinoma (PDAC) is a severe health issue with low survival rates. This study is aimed to improve PDAC treatment by examining the *KRAS* gene mutation, which is common in these tumors. The study involved 193 patients who had surgery for PDAC. Researchers used different sequencing methods to measure the *KRAS* mutation levels and compared these with clinical data. They found that higher *KRAS* mutation levels were linked to faster tumor growth and earlier recurrence after surgery. By combining *KRAS* mutation data with clinical factors like tumor size and a blood marker, they developed a scoring system to predict patient outcomes. This system could help doctors tailor treatments more effectively. The study suggests that using *KRAS* mutation levels can improve predictions about PDAC progression and guide personalized treatment plans in the future.

This summary was initially drafted using artificial intelligence, then revised and fact-checked by the author.

## Introduction

Pancreatic ductal adenocarcinoma (PDAC) is a major global health problem with a dismal prognosis^1,2^. Despite advances in surgery and chemotherapy over the past few decades, the 5-year survival rate of patients with PDAC is only over 10%^3^. This is because image-based diagnosis and nontargeted cytotoxic treatment are still mainly used without an understanding of the molecular profile of patients^4,5^. In breast and lung cancers, for whom survival rates have improved, personalized treatment has already been available depending on the molecular subtype^6,7^. Therefore, a biological understanding is essential for overcoming this devastating disease.

As part of efforts to increase biological understanding, multiomics profiling has been actively conducted in PDAC with improvements in sequencing technologies and computing power^8–11^. However, the vast majority of the results are not applicable to real clinical practice. This is because most multiomics approaches produce complex data and are not cost effective in real-world situations. However, clinical variables are relatively easy to obtain, and their clinical significance can be interpreted through correlations with previous clinical studies. For practical and clinical use of genomics, it is necessary to narrow down the key genes involved in PDAC and combine genomic data with clinical variables.

*KRAS* encodes a GTPase that acts as a molecular switch for various cellular processes^12^. Therefore, *KRAS* mutations are the most common driver mutations in the normal duct epithelium leading to the progression of pancreatic intraepithelial neoplasms^13^. In particular, *KRAS* mutations frequently occur at codon 12, and *KRAS* G12 mutations are reportedly associated with clinical phenotypes^14,15^. However, the correlation between the *KRAS* G12 mutation dosage and clinical phenotype remains unclear^16^. In addition, various mutant dosage ranges have been measured using different sequencing methodologies with varying detection sensitivities, but these have not been compared in relation to clinical outcomes. Therefore, we aimed to investigate the correlation between *KRAS* G12 mutation dosage from various sequencing datasets and clinical phenotypes in patients with PDAC. In addition, a comparison of the transcriptome data between the high- and low-*KRAS*-G12-mutant groups revealed gene expression signatures related to cell cycle activation. Finally, we established a clinically feasible prognostic scoring system by integrating the *KRAS* G12 mutation dosage with clinical information.

## Materials and Methods

### Patient cohort

This single-center, cohort study was approved by the Institutional Review Board of Seoul National University Hospital (H-1705-031-852) and was conducted in compliance with the 1975 Declaration of Helsinki and its later versions. Among patients who underwent pancreatectomy at Seoul National University Hospital between 2009 and 2016, 196 patients with sufficient tumor tissue for next-generation sequencing were included in this study. All samples used in this study were obtained with written informed consent, but participants did not receive compensation. The samples utilized in this study were provided by the biobank at Seoul National University Hospital (blood samples), which is a part of the Korea Biobank Network (KBN4_A03), and Seoul National University Hospital Cancer Tissue Bank (tissue samples). Patients who underwent pancreatectomy with a grossly positive resection margin (*n* = 3) were excluded from the analysis.

All surgical procedures were performed by a team specializing in pancreatic surgery, and tumor samples for next-generation sequencing were collected immediately after resection. Surgical specimens were then prepared and assessed by an experienced gastrointestinal pathologist. All patients were diagnosed with PDAC in the final pathology report. Patients were followed up at the outpatient clinic by either a surgeon or medical oncologist. Following the completion of adjuvant therapy, recurrence was evaluated using carbohydrate antigen (CA) 19-9 levels and computed tomography (CT) of the chest, abdomen, and pelvis every 3 months.

### Estimating tumor purity

To assess tumor purity, qpure was used^17^. Samples from the tumor and matched blood were hybridized to an Infinium Global Screening Array (Illumina) and analyzed through GenomeStudio (version 2) to determine log R-ratio and B-allele frequency values. On the basis of these values, qpure was used to estimate tumor purity levels ranging from 0.15 to 1.

### Analysis of RNA sequencing (RNA-seq) data

RNA exome capture sequencing was performed to overcome RNA degradation problems. After total RNA was quantified via Quant-IT RiboGreen (Thermo Fisher Scientific, Waltham, MA, USA), the proportion of RNA fragments with greater than 200 nucleotides was calculated per sample using TapeStation RNA screenTape (Agilent, Santa Clara, CA, USA). mRNA encoding the exome was isolated from total RNA via the TruSeq RNA exome (Illumina, San Diego, CA, USA). cDNA libraries were synthesized from the fragmented RNA via adapter ligation, and all the libraries were sequenced via the Illumina HiSeq 2500 platform to generate paired-end 101-base pair reads.

The data processing procedures were divided into three main steps. First, Trimmomatic (version 0.36) was used to trim reads with adapter sequences and low-quality bases^18^. Second, STAR (version 2.5.3a) was used to map the refined reads to the human reference genome (hg38)^19^. Third, reads bearing the *KRAS* G12 wild-type and mutant genotypes were directly extracted from the mapped BAM file. The *KRAS* G12 mutant dosage was defined as the mutant read count divided by the total read count.

We used aligned reads to measure the transcriptional levels in samples according to GENCODE v27 GTF annotation via RSEM (version 1.3.0) for the analysis^20^. A count table was used to identify differentially expressed genes (DEGs) in the high- and low-*KRAS*-mutant dosage groups via R/Bioconductor DESeq2 (version 1.40.1)^21^. Significantly upregulated DEGs were used to construct the protein–protein interaction (PPI) networks using STRING^22^. We extracted the interactors of the selected molecules ( > 0.5 STRING score), and the extracted nodes and edges were visualized via Cytoscape (version 3.10.0)^23^.

### Analysis of whole-exome sequencing (WES) data

WES was performed with the SureSelect Human All Exon v5 probe set (Agilent, Santa Clara, CA, USA) according to the Agilent SureSelect Target Enrichment protocol (version B.3, June 2015). Libraries were sequenced with paired-end 101-base pair reads using the Illumina HiSeq 2500 platform. Considering the low tumor purity of PDAC, the sequencing coverage of blood and tumor samples was greater than 100× and 300×, respectively.

The data processing procedures were divided into three main steps. First, BWA (version 0.7.17) was used to map the reads of FASTQ files to hg38^24^. Second, the mapped BAM files were further preprocessed using the Genome Analysis Tool Kit (version 4.1.9)^25^. Third, Mutect2 was used for somatic variant calling by pairing the tumor and blood samples for each patient^26^. The *KRAS* G12 mutant dosage was defined as a variant allele frequency from the VCF file. VCF files annotated with ANNOVAR (version 2018Apr16) were used to generate a mutation profile^27^. The resulting ANNOVAR tables were converted to MAF files and visualized via R/Bioconductor maftools^28^.

### Copy number variation (CNV) analysis

For CNV analysis, segmented copy data were obtained from WES data via a CNV kit (version 0.9.6)^29^. Segmentation data not aligning with the 22 major autosomes or sex chromosomes were excluded. The filtered data were analyzed via GISTIC 2.0 (version 2.0.22) to identify regions and genes with significant copy number changes^30^. Variants were annotated using SnpEff (version 4.3) into missense and nonsense mutations, in-frame insertions/deletions, and frameshift insertions/deletions^31^. Exome coverage was defined as the overlapping genomic regions between the capture probe areas and callable regions using the GATK CallableLoic tool^32^.

### Analysis of *KRAS*-targeted sequencing (TS) data

*KRAS* amplicons were sequenced with > 1,000,000× depth for the G12 position (hg19, chromosome 12: 25,398,283-25,398,285) using customized primers^11^. All the libraries were sequenced using the Illumina NovaSeq platform to generate 151-base pair paired-end reads.

The data processing procedures were divided into 3 main steps. First, Trimmomatic (version 0.36) was employed to filter reads with adapters and low-quality bases^18^. Second, BWA (version 0.7.17) was used to map the filtered reads to the customized *KRAS* reference^24^. Third, reads were extracted from mapped BAM files as previously described for RNA-seq. Furthermore, the *KRAS* G12 mutation dosage was defined as the mutant read count divided by the total read count.

### Thresholds for the high- and low-dose groups

The cutoffs for dividing patients into high- and low-*KRAS*-mutant groups were determined using the log-rank test. For RNA-seq and WES, the most significantly optimal values were employed, whereas two distinct local maxima in the bimodal distribution were utilized for TS (Supplementary Fig. 1).

### Clinical data collection

Clinical variables were extracted from the prospectively collected institutional database. Preoperative information included age, sex, American Society of Anesthesiologists physical status classification, tumor location at baseline CT, maximal tumor diameter at baseline CT, and preoperative CA 19-9 level. The maximal tumor diameter ( ≤ 20 vs. > 20 mm) and CA 19-9 level ( ≤ 150 vs. > 150 U/mL) were converted into categorical variables according to the current American Joint Committee on Cancer 8^th^ edition staging system and previous studies evaluating prognostic factors^33–35^. Follow-up data were also collected to evaluate patient prognosis. Overall survival (OS) was measured from the date of pancreatectomy until the patient’s death or the last visit to the hospital. Recurrence-free survival (RFS) was measured from the date of pancreatectomy until the detection of recurrence or death. With respect to the timing of recurrence, a RFS of less than 6 months was considered very early recurrence; a RFS between 6 and 12 months was considered early recurrence; and a RFS of greater than or equal to 12 months was considered late recurrence^36,37^.

### Statistical analysis

Statistical analysis was performed via R software, version 4.2.1 (R Foundation for Statistical Computing). The maxstat package of R was used to determine the ideal cutoff values for the *KRAS* G12 mutation dosage, which distinguishes OS. To compare clinical variables between the high- and low-mutant dosage groups, the chi-square test and independent t test were used for categorical and continuous variables, respectively. Survival analysis was performed via Kaplan‒Meier curves and the log-rank test. Cox proportional hazards regression models were used to calculate hazard ratios (HRs) and 95% confidence intervals (CIs) of relevant variables. All *P* values were two-sided, and *P* < 0.05 was considered statistically significant.

## Results

### Quantification of the *KRAS* G12 mutation dosage in matched samples via multiple sequencing methods

**Fig. 1:**
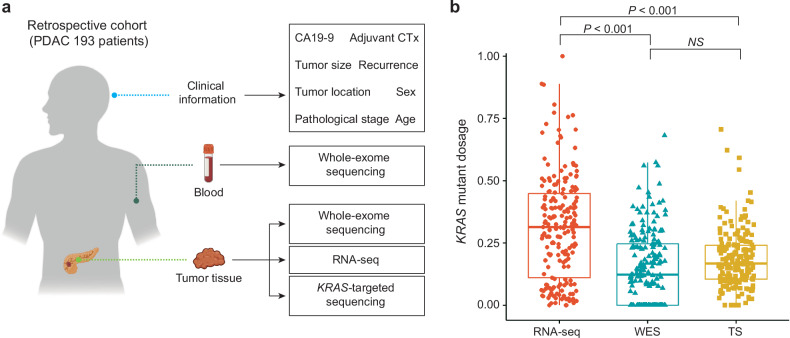
Calculations of the *KRA*S G12 mutation dosage using diverse sequencing methodologies. **a** Overview of WES, RNA-seq, and TS data generated from 193 patients with PDAC. **b** Differences in the *KRAS* mutation dosage according to sequencing method. The *P* value was calculated via an independent t test. NS not significant; RNA-seq RNA sequencing; WES whole-exome sequencing; TS *KRAS*-targeted sequencing.

This retrospective cohort study included 193 PDAC patients who underwent curative-intent pancreatectomy at Seoul National University Hospital between 2009 and 2016. To thoroughly quantify the dosage via multiple sequencing methods along with comprehensive genomic and transcriptomic profiling, the *KRAS* G12 mutation dosage was determined using RNA-seq, WES, and TS simultaneously from the same sample (Fig. 1a). In this study, the *KRAS* mutation dosage represents the variant allele frequency in the WES and TS data, whereas it indicates the number of *KRAS* mutations in the RNA-seq data. The median mutation dosage was significantly greater for RNA-seq than for WES and TS (*P* < 0.001) (Fig. 1b).

### Clinical implications of the *KRAS* mutation level

**Fig. 2:**
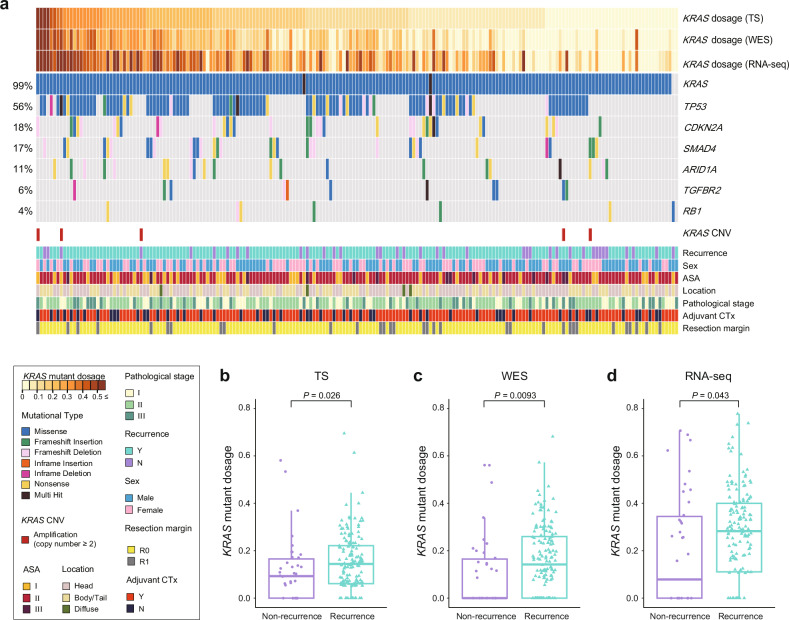
Multiomic profiling of *KRAS* G12 mutation dosage and clinical relevance. **a** Mutation profiles of significantly mutated genes (SMGs) in pancreatic cancer. *KRAS* mutations were determined by combining WES, TS, and RNA-seq data, whereas mutations of six SMGs were extracted from WES data. The samples were sorted according to the *KRAS* dosage calculated from TS data. Patients exhibiting amplifications in the *KRAS* gene are highlighted in red. The bottom panel displays the clinical parameters for each patient. **b**–**d** Comparison of the *KRAS* mutation dosage between patients who experienced recurrence and those who did not. Higher mutation rates were observed in patients with recurrence than in patients without recurrence. The *P* value was calculated using the Wilcoxon signed-rank test. RNA-seq, RNA sequencing; TS *KRAS*-targeted sequencing; WES whole-exome sequencing; ASA American Society of Anesthesiologists; Adjuvant CTx adjuvant chemotherapy.

The mutational landscape of PDAC determined using WES, RNA-seq, and TS revealed mutation frequencies of significantly mutated genes (SMGs) similar to those reported in previous large-scale PDAC genomic studies^10,11,38^. Interestingly, visualization of the clinical information along with the landscape revealed a significant association between recurrence and the *KRAS* mutation level (Fig. 2). Patients who experienced recurrence had higher median mutation dosages (0.144 for TS data, 0.142 for WES data, and 0.283 for RNA-seq data) than patients who did not experience recurrence (0.093 for TS data, 0 for WES data, and 0.079 for RNA-seq data; Fig. 2b-d). These results suggest that the *KRAS* mutation dosage strongly correlates with clinical phenotypes.

To examine the correlation between the *KRAS* G12 mutation dosage and the mutation frequencies of SMGs, we examined six SMGs, including *TP53*, *CDKN2A*, *SMAD4*, *ARID1A*, *TGFBR2*, and *RB1*, using WES data^11^. With increasing *KRAS* mutation dosage, mutations in SMGs tended to be more prevalent (Fig. 2a). Among the 20 patients with *KRAS* mutation dosages above 0.3, 17 (85%) presented mutations in SMGs. In contrast, of the patients with low mutation dosage less than 0.1, only 60.5% (49/81) had mutations in SMGs. Despite the high chance of SMG mutations in the high-dose group, no obvious statistical association was noted between clinical information and the mutation frequencies of the six SMGs (Supplementary Fig. 2, Supplementary Table 1). Thus, *KRAS* has particularly significant clinical implications compared with the other SMGs.

**Fig. 3:**
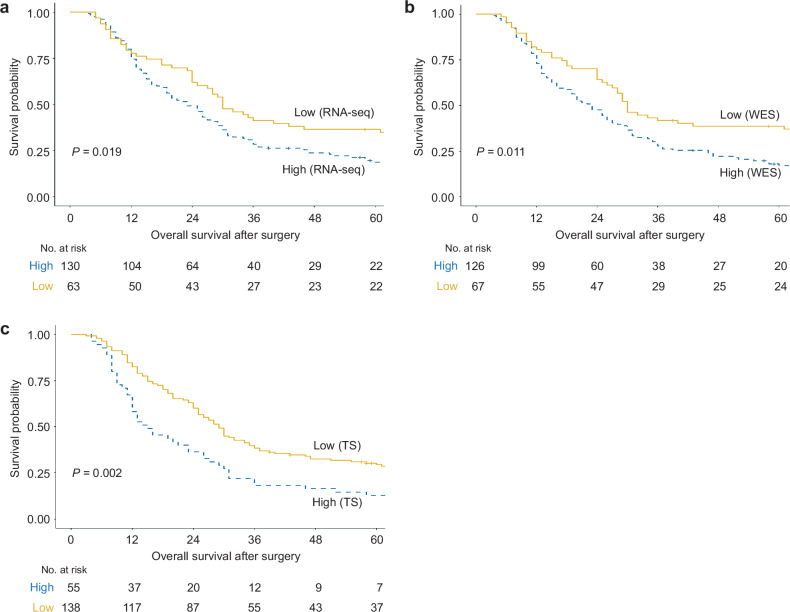
Comparison of overall survival between patients with high and low *KRAS* mutation dosages. **a** Using TS data, the low-mutant-dose group (29.0 months, 95% CI, 15.0–72.0 months) had a longer median OS than did the high-mutant-dose group (15.0 months, 95% CI, 9.0–31.0 months). **b** Using WES data, the median OS was longer in the low-mutant-dose group (30.0 months, 95% CI, 17.0–97.0 months) than in the high-mutant-dose group (23.0 months, 95% CI, 12.0–46.0 months). **c** RNA-seq data revealed that patients treated with a low mutation dosage (30.0 months, 95% CI, 15.0–99.0 months) presented a longer median OS than did those treated with a high mutation dosage (23.0 months, 95% CI, 12.0–47.0 months). Survival analysis was performed via Kaplan‒Meier curves and the log-rank test. RNA-seq RNA sequencing; WES whole-exome sequencing; TS *KRAS*-targeted sequencing.

We divided patients into high- and low-*KRAS*-mutant groups and compared their baseline clinical characteristics (Table 1). Groups were defined differently according to sequencing methods; 63 patients (32.6%, dosage <0.163), 67 patients (34.7%, no *KRAS* G12 mutation), and 138 patients (71.5%, dosage <0.195) were assigned to the low-mutant dosage group according to RNA-seq, WES, and TS data, respectively. Compared with the high-dose group, the low-dose group presented a longer median OS (Fig. 3). In particular, TS data showed the greatest difference in OS between the mutant dosage groups (29.0 months, 95% CI, 15.0–72.0 months in the low-dose group; 15.0 months, 95% CI, 9.0–31.0 months in the high-dose group; *P* = 0.002; Fig. 3c). Moreover, the mutant dosage calculated from TS data exhibited the most clinical relevance. For example, TS showed a high proportion of patients with larger tumors ( > 20 mm) at baseline CT in the high-dose mutant group (89.1%) than in the low-dose mutant group (73.2%; *P* = 0.021). With respect to recurrence timing, TS revealed that more patients with a low mutation dosage (44.8%) experienced late recurrence than did those with a high mutation dosage (22.0%; *P* = 0.007). Notably, half of the patients with high mutation dosages experienced very early recurrence within six months after surgery, and the mutation dosage determined using TS data was also significantly inversely correlated with RFS (Table 2). These results suggest that TS data provided the most clinically relevant mutation dosage compared with RNA-seq and WES data.

**Table 1:** Baseline clinical characteristics according to the *KRAS* G12 mutation dosage.

Variables	Total	High	Low	*P* value	High	Low	*P* value	High	Low	*P* value
		(RNA-seq)	(RNA-seq)		(WES)	(WES)		(TS)	(TS)	
N (%)	193	130 (67.4)	63 (32.6)		126 (65.3)	67 (34.7)		55 (28.5)	138 (71.5)	
Age, mean (SD), y	64.7 (10.2)	65.1 (9.9)	64.0 (10.8)	0.515	64.7 (10.4)	64.9 (9.8)	0.909	64.9 (9.7)	64.7 (10.4)	0.907
Sex				1.000			0.446			0.524
Male	109 (56.5)	73 (56.2)	36 (57.1)		74 (58.7)	35 (52.2)		29 (52.7)	80 (58.0)	
Female	84 (43.5)	57 (43.8)	27 (42.9)		52 (41.3)	32 (47.8)		26 (47.3)	58 (42.0)	
ASA classification				0.775			0.565			0.357
I/II	179 (92.7)	121 (93.1)	58 (92.1)		118 (93.7)	61 (91.0)		53 (96.4)	126 (91.3)	
III/IV	14 (7.3)	9 (6.9)	5 (7.9)		8 (6.3)	6 (9.0)		2 (3.6)	12 (8.7)	
Location				0.065			0.183			1.000
Head	109 (56.5)	70 (53.8)	39 (61.9)		70 (55.6)	39 (58.2)		31 (56.4)	78 (56.5)	
Body/Tail	80 (41.5)	59 (45.4)	21 (33.3)		55 (43.7)	25(37.3)		23 (41.8)	57 (41.3)	
Diffuse	4 (2.0)	1 (0.8)	3 (4.8)		1 (0.8)	3 (4.5)		1 (1.8)	3 (2.2)	
Tumor size, mm				0.716			1.000			0.021
> 20	150 (77.7)	102 (78.5)	48 (76.2)		98 (77.8)	52 (77.6)		49 (89.1)	101 (73.2)	
≤ 20	43 (22.3)	28 (21.5)	15 (23.8)		28 (22.2)	15 (22.4)		6 (10.9)	37 (26.8)	
CA 19-9 level, U/mL				0.092			0.050			1.000
> 150	91 (47.2)	67 (51.5)	24 (38.1)		66 (52.4)	25 (37.3)		26 (47.3)	65 (47.1)	
≤ 150	102 (52.8)	63 (48.5)	39 (61.9)		60 (47.6)	42 (62.7)		29 (52.7)	73 (52.9)	
Pathological stage				0.124			0.225			0.458
I	55 (28.5)	33 (25.4)	22 (34.9)		33 (26.2)	22 (32.8)		14 (25.5)	41 (29.7)	
II	97 (50.3)	72 (55.4)	25 (39.7)		69 (54.8)	28 (41.8)		26 (47.3)	71 (51.4)	
III	41 (21.2)	25 (19.2)	16 (25.4)		24 (19.0)	17 (25.4)		15 (27.2)	26 (18.8)	
Resection margin				0.222			0.074			0.399
R0	160 (82.9)	111 (85.4)	49 (77.8)		109 (86.5)	51 (76.1)		48 (87.3)	112 (81.2)	
R1	33 (17.1)	19 (14.6)	14 (22.2)		17 (13.5)	16 (23.9)		7 (12.7)	26 (18.8)	
Adjuvant CTx				0.867			0.744			0.391
Y	135 (69.9)	90 (69.2)	45 (71.4)		87 (69.0)	48 (71.6)		36 (65.5)	99 (71.7)	
N	58 (30.1)	40 (30.8)	18 (28.6)		39 (31.0)	19 (28.3)		19 (34.5)	39 (28.3)	

*ASA* American Society of Anesthesiologists, *CA 19-9* carbohydrate antigen 19-9, *CTx* chemotherapy, *RNA-seq* RNA sequencing, *TS* *KRAS* targeted sequencing, *WES* whole-exome sequencing.

**Table 2:** Recurrence-related factors according to the *KRAS* G12 mutation dosage.

Variables	High	Low	*P* value	High	Low	*P* value	High	Low	*P* value
	(RNA-seq)	(RNA-seq)		(WES)	(WES)		(TS)	(TS)	
Recurrence			0.006			0.004			0.026
Y	112 (86.2)	43 (68.3)		109 (86.5)	46 (68.7)		50 (90.9)	105 (76.1)	
N	18 (13.8)	20 (31.7)		17 (13.5)	21 (31.3)		5 (9.1)	33 (23.9)	
Recurrence pattern^a^			0.283			1.000			0.299
Local	22 (19.6)	12 (27.9)		24 (22.0)	10 (21.7)		8 (16.0)	26 (24.8)	
Distant	90 (80.4)	31 (72.1)		85 (78.0)	36 (78.3)		42 (84.0)	79 (75.2)	
Recurrence timing^a^			0.231			0.683			0.007
Very early	37 (33.0)	16 (37.2)		37 (33.9)	16 (24.8)		25 (50.0)	28 (26.6)	
Early	36 (32.2)	8 (18.6)		33 (30.3)	11 (23.9)		14 (28.0)	30 (28.6)	
Late	39 (34.8)	19 (44.2)		39 (35.8)	19 (41.3)		11 (22.0)	47 (44.8)	

*RNA-seq* RNA sequencing, *TS* *KRAS*-targeted sequencing, *WES* whole-exome sequencing.

^a^Only reported for patients who experienced recurrence.

### High *KRAS* mutation dosage accelerate tumor progression

**Fig. 4:**
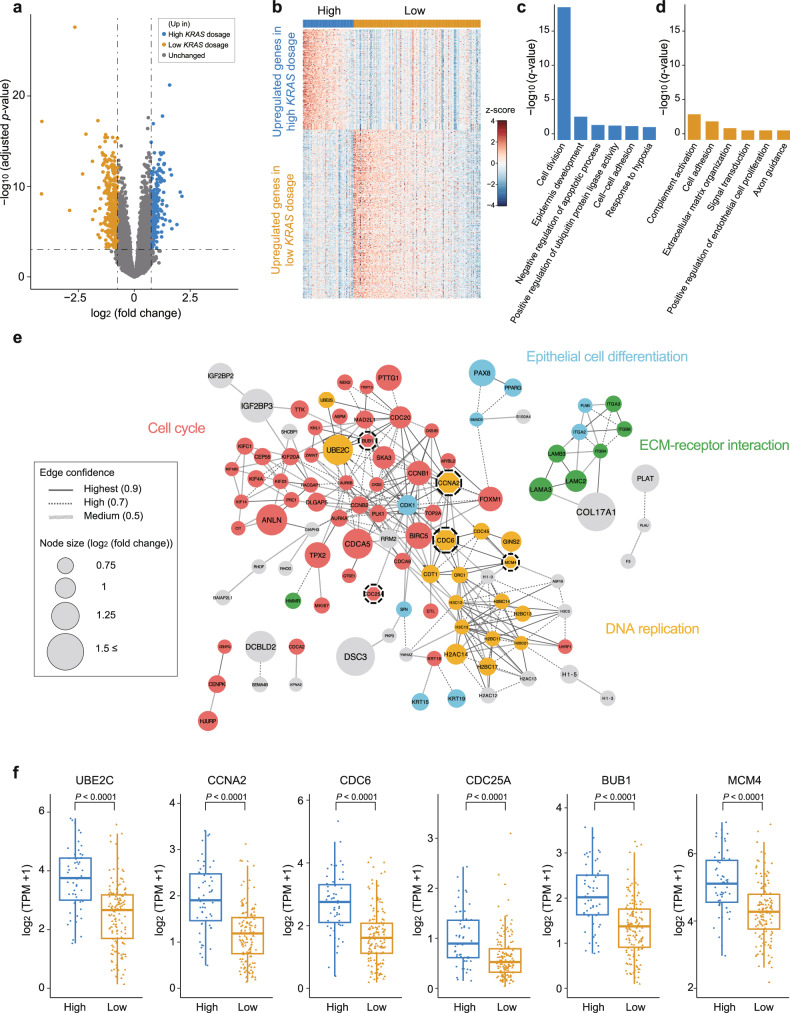
Alteration of transcriptome profiles in tissues according to *KRAS* mutation dosage. **a** Volcano plot showing differentially expressed genes (DEGs) between the high-*KRAS*-dose group and the low-*KRAS*-dose group. Significant DEGs satisfied the following criteria: -log_10_ (adjusted *p* value) <0.05 and an absolute value of log_2_ (fold change) ≥ 0.75. **b** Heatmap of 521 significant DEGs between the high- and low-*KRAS*-dose groups. **c**–**d** Gene Ontology (GO) enrichment analysis of DEGs upregulated in the high-*KRAS* dosage group or low-*KRAS* dosage group. **e** Network model describing interactions of DEGs upregulated in the high-*KRAS*-mutant group. The size of the circles (nodes) and thickness of the lines (edges) are proportional to log_2_(fold change) and the degree of protein‒protein interaction, respectively. Genes are color-coded according to their biological functions. Nodes with dashed lines are genes involved in the RAS pathway. **f** Expression levels of *UBE2C* and RAS pathway genes (*CCNA2*, *CDC6*, *CDC25A*, *BUB1*, and *MCM4*) in the high- and low-dose mutant groups. The high-dose mutant group presented higher expression levels of these genes than did the low-dose mutant group. The *P* value was calculated via Student’s t test. High indicates the high-*KRAS*-mutant dosage group; low indicates the low-*KRAS*-mutant dosage group; ECM extracellular matrix; TPM transcripts per million.

To understand the biological characteristics of the tissues according to the mutation dosage, we compared the transcriptomic data between the high- and low-dose mutant groups determined by TS. Differentially expressed gene (DEG) analysis revealed that 194 and 327 genes were upregulated in the high- and low-dose mutant groups, respectively (Fig. 4a). The upregulated DEGs were able to distinguish the high-dose group from the low-dose group (Fig. 4b). The functional analysis of the DEGs revealed a significant increase in cell division and epidermis development processes within the high-dose mutant group (Fig. 4c). Notably, 16.5% (32/194) of the DEGs were related to cell division. Additionally, DEG network analysis results showed strong genetic interactions between genes involved in the cell cycle and DNA replication (Fig. 4e). The downstream genes related to the RAS pathway, including *CCNA2*, *CDC6, CDC25A, BUB1*, and *MCM4*, were significantly upregulated in the high-dose group (median *CCNA2* 1.90, *CDC6* 2.75, *CDC25A* 0.895, *BUB1* 2.02, *MCM4* 5.11 in the high-dose group; median *CCNA2* 1.19, *CDC6* 1.61, *CDC25A* 0.526, *BUB1* 1.37, *MCM4* 4.28 in the low-dose group; Fig. 4f). Furthermore, the high-dose group presented high expression levels of *UBE2C*, which induces the growth of tumors harboring the *KRAS* G12 mutation^39^, acting as one of the hubs for the cell cycle module (median 3.75 in the high-dose group; 2.67 in the low-dose group; Fig. 4e-f). On the other hand, the low-dose mutant group presented high expression of genes related to complement activation and axonogenesis, with weak statistical significance (Fig. 4d). These genes are mainly enriched in the immune system and immune cell migration (Supplementary Fig. 3). These results indicate that a high dosage of the *KRAS* mutant can aggravate clinical outcomes by facilitating tumor growth and progression.

### Comparison of survival prediction between the *KRAS* G12 mutation dosage and clinical variables

**Fig. 5:**
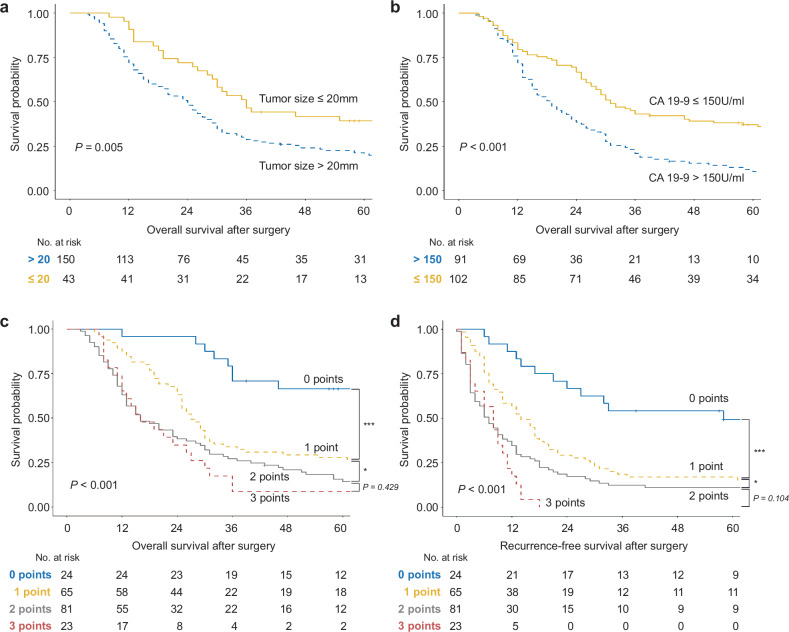
Scoring system for survival prediction by *KRAS* G12 mutation dosage and clinical variables. **a**‒**b** OS according to the maximal tumor diameter at baseline CT (HR 0.56; 95% CI 0.38‒0.84; *P* = 0.005 for OS, HR 0.55; 95% CI 0.37‒0.82; *P* = 0.003 for RFS) and the preoperative CA 19‒9 level (HR 0.53; 95% CI 0.38‒0.72; *P* < 0.001 for OS, HR 0.47; 95% CI 0.35‒0.65; *P* < 0.001 for RFS). **c**–**d** OS and RFS according to the prognostic score obtained by integrating the *KRAS* G12 mutation dosage obtained from *KRAS*-targeted sequencing with clinical variables. Survival analysis was performed via Kaplan‒Meier curves and the log-rank test. *, *P* < 0.05; **, *P* < 0.01; ***, *P* < 0.001 according to the log-rank test. CA 19-9, carbohydrate.

To expand the utility of the mutation dosage as a prognostic value, we explored candidate variables for use in a prognostic scoring system by performing Cox proportional hazards regression analysis with clinical variables (Supplementary Table 2). Among the statistically significant variables, maximal tumor diameter at baseline CT ≤ 20 mm (HR 0.56; 95% CI 0.38–0.84; *P* = 0.005 for OS, HR 0.55; 95% CI 0.37–0.82; *P* = 0.003 for RFS) and CA 19–9 levels ≤ 150 U/mL (HR 0.53; 95% CI 0.38–0.72; *P* < 0.001 for OS, HR 0.47; 95% CI 0.35–0.65; *P* < 0.001 for RFS) could be determined before surgery; hence, we selected these variables as candidates for a prognostic scoring system. The preoperative tumor size and CA 19-9 level were similar to those of the *KRAS* G12 mutation dosage for OS prediction (Fig. 5a-b). These results suggest that prognostic power can be improved by integrating genomic and clinical variables with similar performance in predicting survival.

### Scoring system for survival prediction based on *KRAS* G12 mutation dosage and clinical variables

We established a scoring system by integrating three variables: maximal tumor diameter at baseline CT, CA 19-9 level, and *KRAS* G12 mutation dosage based on TS data. As per the devised categorical rating system, patients were given 1 point each for maximal tumor diameter at baseline CT > 20 mm, preoperative CA 19-9 levels > 150 U/mL, and *KRAS* G12 mutation dosage based on TS data > 0.195. All patients received a score ranging from a minimum of 0 to a maximum of 3 and were stratified by the prognostic score. Among the 193 patients, 24 (12.4%), 65 (33.7%), 81 (42.0%), and 23 (11.9%) scored 0, 1, 2, and 3 points, respectively. To evaluate the performance of this prognostic scoring system, we assessed OS and RFS for patients according to the prognostic scores (Fig. 5c,d, Table 3). Superior OS and RFS were observed in patients with 0 points, with gradual worsening noted as the points increased. We also found that the higher the prognostic score was, the significantly greater the HRs for OS and RFS. Patients with 0 points had superior median OS (RFS) of 97.0 (58.0) months and the 1-year, 3-year, and 5-year OS (RFS) rates were 97.0 (58.0) months, 95.8% (87.5%), 70.8% (54.2%), and 66.4% (49.2%), respectively. In contrast, patients with 3 points had a significantly worse median OS (RFS) of only 16.0 (8.0) months and the 1-year, 3-year, and 5-year OS (RFS) rates were 65.2% (17.4%), 8.7% (0.0%), and 8.7% (0.0%), respectively. In addition, for patients with scores of 0 and 1 point, 25.0% (3/12) and 51.0% (26/51) of patients, respectively, experienced very early or early recurrence, whereas 73.9% (68/92) of patients with scores of 2 or 3 points experienced very early or early recurrence (Supplementary Fig. 4).

**Table 3:** Correlations of the prognostic scoring system with overall and recurrence-free survival outcomes.

			Overall Survival			
Scores (% of patients)	HR (95% CI)	*P* value	Median, month	1 year	3 year	5 year
0 points (12.4%)	1.00 (Reference)	NA	97.0	95.8%	70.8%	66.4%
1 point (33.7%)	2.78 (1.45, 5.33)	0.002	27.0	87.7%	33.8%	27.7%
2 points (42.0%)	4.03 (2.13, 7.62)	<0.001	16.0	63.0%	27.2%	14.3%
3 points (11.9%)	5.04 (2.43, 10.45)	<0.001	16.0	65.2%	8.7%	8.7%

			Recurrence-Free Survival			
Scores (% of patients)	HR (95% CI)	*P* value	Median, month	1 year	3 year	5 year
0 points (12.4%)	1.00 (Reference)	NA	58.0	87.5%	54.2%	49.2%
1 point (33.7%)	2.62 (1.43, 4.79)	0.002	14.0	56.9%	18.5%	16.9%
2 points (42.0%)	3.94 (2.18, 7.13)	<0.001	7.0	34.6%	12.4%	11.1%
3 points (11.9%)	6.59 (3.27, 13.30)		8.0	17.4%	0.0%	0.0%

*CI* confidence interval, *HR* hazard ratio, *NA* not applicable.

The prognostic power of data from TS were superior to RNA-seq and WES data in a scoring system when the mutation dosages were integrated with clinical variables. In a scoring system with a single clinical variable, statistically significant differences in OS and RFS were noted between patients with 0 points and those with 1 point (Supplementary Fig. 5a,b). The addition of the mutant dosage obtained from RNA-seq data mitigated differences in OS and RFS between patients with 0 points and those with 1 point according to the clinical variable score, but these differences were maintained when the mutant dosage was obtained using WES or TS data (Supplementary Fig. 5c–f, Fig. 5c, d). Interestingly, with the inclusion of the mutant dosage from TS, all patients with 3 points experienced recurrence within 2 years (Fig. 5d), indicating that the integration of the mutant dosage allowed for the identification of the exceptionally poor prognostic group. In contrast, the mutant dosage obtained from RNA-seq and WES data marginally contributed to differences in OS and RFS between patients with 2 points and those with 3 points in contrast that obtained using TS. Taken together, these findings indicate that the *KRAS* mutation dosage derived from TS data improved the prognostic power of existing clinical variables.

## Discussion

Molecular PDAC subtypes were successfully established through these studies, and their prognostic value was confirmed^8–11^. However, these results remain on the bench owing to high data complexity and low cost-effectiveness. In this study, we resolved this issue by exclusively utilizing the *KRAS* G12 mutation along with preoperative clinical variables to evaluate PDAC prognosis, resulting in a clinically feasible prognostic scoring system.

To determine the threshold value for high and low dosage classification, two optimal values were identified for TS through the log-rank test: 0.139 (log-rank test *p* value = 0.0019) and 0.195 (log-rank test *p* value = 0.002) (Supplementary Fig. 1). Both cutoffs yielded reproducible conclusions in genomic analysis and clinical interpretation (Supplementary Figs. 6-9, Supplementary Table 3). A threshold of 0.195 was used for the main figures because it has slightly better predictive power, and supplementary data included all the analysis results using a cutoff of 0.139 (Supplementary Fig. 10, Supplementary Tables 4, 5).

The *KRAS* G12 mutation has been used as a clinically applicable variable given its high frequency and targetability in PDAC^14,15^. However, the prognostic power of mutation dosage remains unclear. In our improved prognostic scoring system, the mutation dosage variable could contribute to the prognostic value of clinical variables as a molecular measurement of tumor purity and/or tumor size because the dosage is likely correlated with purity rather than size (Supplementary Fig. 11d). A comparison of the purity of the microarray data between the high- and low-dose mutant groups revealed that the mutation dose was positively correlated with tumor purity (Supplementary Fig. 11). Notably, TS data yielded the greatest difference in purity between the high- and low-dose groups among the sequencing methods, which is consistent with the superior performance of TS for prognosis. Because the mutation dosage obtained using TS data represents the variant allele frequency of the *KRAS* mutation, it is significantly associated with tumor purity. Another rationale for the relevance of the *KRAS* mutation dose and tumor purity is that *KRAS*^G12mut^ regulates cell proliferation by activating the RAS signaling pathway. For example, in patients with a high *KRAS* mutation dosage, there is increased expression of key regulators of DNA replication and the cell cycle, such as *CCNA2* and *CDC6*, inducing rapid cell proliferation. Additionally, *UBE2C*, a gene required for mutant *KRAS*-induced lung tumorigenesis and associated with malignant transformation^39^, was highly upregulated in the high-dose mutant group, further increasing malignant cell growth.

The *KRAS* mutation dosage has greater clinical relevance than the *KRAS* mutation copy number and tumor cellularity, which are correlated with dosage. High-level amplifications ( ≥ 2 copies) of *KRAS* were detected in five (2.6%) patients, whereas no patient exhibited homozygous *KRAS* deletions (Fig. 2a). Consistent with our data, analyzes of pancreatic adenocarcinoma in The Cancer Genome Atlas (TCGA) and Australian Pancreatic Genome Initiative (APGI) revealed that copy number variations of the *KRAS* gene were only attributed to amplification, with a low incidence^38,40^.

When the *KRAS* mutation dosage was adjusted for CNVs, a marginal change in survival differences between dosage groups by CNV adjustment was observed, leading to the same interpretation of the clinical data (Supplementary Fig. 12). In addition, the *KRAS* mutation dose demonstrated a stronger clinical association than did tumor cellularity. When survival was compared between the *KRAS* mutation dosage groups with similar tumor purity levels, patients with a high *KRAS* mutation dosage experienced shorter RFS than those with a low dosage (Supplementary Fig. 13). These findings indicate that the mutation dosage provides significant prognostic power compared with CNVs and tumor purity.

PDAC is a type of cancer with low tumor purity (median tumor purity determined using microarray data, 0.265). The biologically low tumor purity of PDAC and technically inconsistent variant calling for TS data pose analytical difficulties^41,42^. To overcome the low sensitivity in detecting mutations in cancers with low tumor purity, TS is an optimal method among available sequencing technologies^43,44^. The *KRAS* G12 mutation detection rate was similar across the sequencing technologies in the samples with high tumor purity ( > 50th percentile) (RNA-seq, 78.1%; WES, 79.2%; TS, 83.3%). However, for samples with low tumor purity, TS showed superior detection sensitivity (RNA-seq, 67.0%; WES, 51.5%; TS, 75.3%). These results suggest that TS is a practical and accurate method for quantifying the correlation between mutation dosage and the proportions of malignant cells.

We found that high *KRAS* G12 mutation dosage from the TS data were strongly associated with high recurrence rates and short time intervals from surgery to recurrence. Given that recurrence, especially within 12 months, is associated with poor prognosis, many clinicians have attempted to prevent recurrence after surgery^36^. In addition to recommending adjuvant chemotherapy after surgery for all patients, many studies evaluating the effect of neoadjuvant chemotherapy on recurrence have been conducted^45,46^. According to a recent meta-analysis, neoadjuvant chemotherapy can reduce overall recurrence rates and delay the timing of recurrence in patients with PDAC^47^. Therefore, predicting the likelihood and timing of recurrence at the time of diagnosis is an important medical issue that can affect the overall treatment strategy. On the basis of the findings of our study, we can predict the occurrence and timing of recurrence and develop an individualized treatment strategy for each patient by performing TS and using existing CT and CA 19-9 level data.

Most *KRAS* mutations occur at codon 12, and clonal mutations are prevalent. In the pancreatic adenocarcinoma dataset of TCGA consortium, 131 of 140 patients harboring *KRAS* mutations were clonal (*KRAS*^G12mut^), whereas others (9/140; 6.4%) had subclonal mutations at codons 12 and 61^38^. In this study, 7.3% (14/191) of the patients had subclonal mutations (*KRAS*^G12mut/Q61mut^), whereas the remaining 92.7% (177/191) had clonal mutations (*KRAS*^G12mut^) (Supplementary Table 6). All patients with subclonal mutations were in the low-*KRAS*-mutant subgroup. When the *KRAS* Q61 mutation dosage was considered for the classification of high- and low-dosage groups, four patients with high Q61 VAF values (> 0.195) experienced transitions in group membership, resulting in a more significant survival difference (Supplementary Fig. 14). Therefore, although *KRAS*^G12mut^ is used in this study to represent the mutation dosage, additional subclonal mutations could improve the scoring system.

This study has several limitations. First, the *KRAS* mutation dosage obtained from RNA-seq was computed exclusively on the basis of read counts, without considering biological factors such as posttranscriptional editing. Although the RNA-seq-based dosage included various confounding factors, its significance originated from the dosage directly preceding translation. Second, our results were exclusively based on samples from patients with PDAC who underwent resection; therefore, selection bias was unavoidable. Third, although consistent sequencing results between preoperative biopsy samples and surgical samples have been reported, verification of biopsy samples with TS will be needed for a prognostic scoring system that is completely based on preoperative variables. Finally, validation of the prognostic scoring system for an independent cohort is needed prior to clinical use. Therefore, follow-up studies should be conducted with endoscopic ultrasound biopsy samples from patients with unresectable PDAC to ensure a more versatile application.

In conclusion, our research indicates that the *KRAS* G12 mutation dosage obtained from the TS data is a reliable prognostic factor for PDAC, with comparable predictive power to that of other clinical variables. Additionally, high *KRAS* mutation rates were associated with the overexpression of genes related to the cell cycle. Furthermore, we established a prognostic scoring system that can predict survival outcomes and is highly feasible because it integrates preoperative clinical variables and the *KRAS* mutation dosage from the TS.

## Availability of data and materials

The RNA and whole-exome sequencing data used in this study are available from dbGaP (https://www.ncbi.nlm.nih.gov/gap/; accession ID: phs002347.v1.p1).

## Supplementary information

Supplementary Information

## References

[1] Park, W., Chawla, A. & O’Reilly, E. M. Pancreatic cancer: a review. *JAMA* **326**, 851–862 (2021). 34547082 10.1001/jama.2021.13027 PMC9363152

[2] Rahib, L., Wehner, M. R., Matrisian, L. M. & Nead, K. T. Estimated projection of US cancer incidence and death to 2040. *JAMA Netw. Open* **4**, e214708 (2021). 33825840 10.1001/jamanetworkopen.2021.4708 PMC8027914

[3] Siegel, R. L., Miller, K. D., Fuchs, H. E. & Jemal, A. Cancer statistics, 2022. *CA Cancer J. Clin.* **72**, 7–33 (2022). 35020204 10.3322/caac.21708

[4] Takikawa, T. et al. Clinical features and prognostic impact of asymptomatic pancreatic cancer. *Sci. Rep.* **12**, 4262 (2022). 35277545 10.1038/s41598-022-08083-6 PMC8917162

[5] Tempero, M. A. et al. Pancreatic adenocarcinoma, Version 2.2021, NCCN Clinical Practice Guidelines in Oncology. *J. Natl Compr. Canc Netw.* **19**, 439–457 (2021). 33845462 10.6004/jnccn.2021.0017

[6] Gradishar, W. J. et al. Breast cancer, Version 3.2022, NCCN Clinical Practice Guidelines in Oncology. *J. Natl Compr. Canc Netw.* **20**, 691–722 (2022). 35714673 10.6004/jnccn.2022.0030

[7] Ettinger, D. S. et al. Non-small cell lung cancer, Version 3.2022, NCCN Clinical Practice Guidelines in Oncology. *J. Natl Compr. Canc Netw.* **20**, 497–530 (2022). 35545176 10.6004/jnccn.2022.0025

[8] Collisson, E. A. et al. Subtypes of pancreatic ductal adenocarcinoma and their differing responses to therapy. *Nat. Med*. **17**, 500–503 (2011). 21460848 10.1038/nm.2344 PMC3755490

[9] Moffitt, R. A. et al. Virtual microdissection identifies distinct tumor- and stroma-specific subtypes of pancreatic ductal adenocarcinoma. *Nat. Genet* **47**, 1168–1178 (2015). 26343385 10.1038/ng.3398 PMC4912058

[10] Bailey, P. et al. Genomic analyses identify molecular subtypes of pancreatic cancer. *Nature* **531**, 47–52 (2016). 26909576 10.1038/nature16965

[11] Hyeon, D. Y. et al. Proteogenomic landscape of human pancreatic ductal adenocarcinoma in an Asian population reveals tumor cell-enriched and immune-rich subtypes. *Nat. Cancer* **4**, 290–307 (2023). 36550235 10.1038/s43018-022-00479-7

[12] Buscail, L., Bournet, B. & Cordelier, P. Role of oncogenic KRAS in the diagnosis, prognosis and treatment of pancreatic cancer. *Nat. Rev. Gastroenterol. Hepatol.* **17**, 153–168 (2020). 32005945 10.1038/s41575-019-0245-4

[13] Hosein, A. N., Dougan, S. K., Aguirre, A. J. & Maitra, A. Translational advances in pancreatic ductal adenocarcinoma therapy. *Nat. Cancer* **3**, 272–286 (2022). 35352061 10.1038/s43018-022-00349-2

[14] Qian et al. Association of alterations in main driver genes with outcomes of patients with resected pancreatic ductal adenocarcinoma. *JAMA Oncol.* **4**, e173420 (2018). 29098284 10.1001/jamaoncol.2017.3420 PMC5844844

[15] Guo, S. et al. Preoperative detection of KRAS G12D mutation in ctDNA is a powerful predictor for early recurrence of resectable PDAC patients. *Br. J. Cancer* **122**, 857–867 (2020). 31969677 10.1038/s41416-019-0704-2 PMC7078253

[16] Mueller, S. et al. Evolutionary routes and KRAS dosage define pancreatic cancer phenotypes. *Nature* **554**, 62–68 (2018). 29364867 10.1038/nature25459 PMC6097607

[17] Song, S. et al. qpure: A tool to estimate tumor cellularity from genome-wide single-nucleotide polymorphism profiles. *PLoS One* **7**, e45835 (2012). 23049875 10.1371/journal.pone.0045835 PMC3457972

[18] Bolger, A. M., Lohse, M. & Usadel, B. Trimmomatic: a flexible trimmer for Illumina sequence data. *Bioinformatics* **30**, 2114–2120 (2014). 24695404 10.1093/bioinformatics/btu170 PMC4103590

[19] Dobin, A. et al. STAR: ultrafast universal RNA-seq aligner. *Bioinformatics* **29**, 15–21 (2013). 23104886 10.1093/bioinformatics/bts635 PMC3530905

[20] Li, B. & Dewey, C. N. RSEM: accurate transcript quantification from RNA-Seq data with or without a reference genome. *BMC Bioinforma.* **12**, 323 (2011). 10.1186/1471-2105-12-323 PMC3163565 21816040

[21] Love, M. I., Huber, W. & Anders, S. Moderated estimation of fold change and dispersion for RNA-seq data with DESeq2. *Genome Biol.* **15**, 550 (2014). 25516281 10.1186/s13059-014-0550-8 PMC4302049

[22] Szklarczyk, D. et al. STRING v10: protein-protein interaction networks, integrated over the tree of life. *Nucleic Acids Res.* **43**, D447–D452 (2015). 25352553 10.1093/nar/gku1003 PMC4383874

[23] Shannon, P. et al. Cytoscape: a software environment for integrated models of biomolecular interaction networks. *Genome Res.* **13**, 2498–2504 (2003). 14597658 10.1101/gr.1239303 PMC403769

[24] Li, H. & Durbin, R. Fast and accurate long-read alignment with Burrows-Wheeler transform. *Bioinformatics* **26**, 589–595 (2010). 20080505 10.1093/bioinformatics/btp698 PMC2828108

[25] Van der Auwera, G. A. et al. From FastQ data to high confidence variant calls: the genome analysis toolkit best practices pipeline. *Curr. Protoc. Bioinforma.* **43**, 11 10 11–11 10 33 (2013). 10.1002/0471250953.bi1110s43 PMC4243306 25431634

[26] Cibulskis, K. et al. Sensitive detection of somatic point mutations in impure and heterogeneous cancer samples. *Nat. Biotechnol.* **31**, 213–219 (2013). 23396013 10.1038/nbt.2514 PMC3833702

[27] Wang, K., Li, M. & Hakonarson, H. ANNOVAR: functional annotation of genetic variants from high-throughput sequencing data. *Nucleic Acids Res.* **38**, e164 (2010). 20601685 10.1093/nar/gkq603 PMC2938201

[28] Mayakonda, A., Lin, D. C., Assenov, Y., Plass, C. & Koeffler, H. P. Maftools: efficient and comprehensive analysis of somatic variants in cancer. *Genome Res*. **28**, 1747–1756 (2018). 30341162 10.1101/gr.239244.118 PMC6211645

[29] Talevich, E., Shain, A. H., Botton, T. & Bastian, B. C. CNVkit: genome-wide copy number detection and visualization from targeted DNA sequencing. *PLoS Comput Biol.* **12**, e1004873 (2016). 27100738 10.1371/journal.pcbi.1004873 PMC4839673

[30] Mermel, C. H. et al. GISTIC2.0 facilitates sensitive and confident localization of the targets of focal somatic copy-number alteration in human cancers. *Genome Biol.* **12**, R41 (2011). 21527027 10.1186/gb-2011-12-4-r41 PMC3218867

[31] Cingolani, P. et al. A program for annotating and predicting the effects of single nucleotide polymorphisms, SnpEff: SNPs in the genome of Drosophila melanogaster strain w1118; iso-2; iso-3. *Fly. (Austin)* **6**, 80–92 (2012). 22728672 10.4161/fly.19695 PMC3679285

[32] McKenna, A. et al. The genome analysis toolkit: a MapReduce framework for analyzing next-generation DNA sequencing data. *Genome Res.* **20**, 1297–1303 (2010). 20644199 10.1101/gr.107524.110 PMC2928508

[33] Connor, S. et al. Serum CA19-9 measurement increases the effectiveness of staging laparoscopy in patients with suspected pancreatic malignancy. *Dig. Surg.* **22**, 80–85 (2005). 15849467 10.1159/000085297

[34] Halloran, C. M. et al. Carbohydrate antigen 19.9 accurately selects patients for laparoscopic assessment to determine resectability of pancreatic malignancy. *Br. J. Surg.* **95**, 453–459 (2008). 18161888 10.1002/bjs.6043

[35] Crippa, S. et al. Implications of perineural invasion on disease recurrence and survival after pancreatectomy for pancreatic head ductal adenocarcinoma. *Ann. Surg.* **276**, 378–385 (2022). 33086324 10.1097/SLA.0000000000004464

[36] Groot, V. P. et al. Patterns, timing, and predictors of recurrence following pancreatectomy for pancreatic ductal adenocarcinoma. *Ann. Surg.* **267**, 936–945 (2018). 28338509 10.1097/SLA.0000000000002234

[37] Daamen, L. A. et al. Preoperative predictors for early and very early disease recurrence in patients undergoing resection of pancreatic ductal adenocarcinoma. *HPB (Oxf.)* **24**, 535–546 (2022). 10.1016/j.hpb.2021.09.004 34642090

[38] Cancer Genome Atlas Research Network. Electronic address aadhe, Cancer Genome Atlas Research N. Integrated genomic characterization of pancreatic ductal adenocarcinoma. *Cancer Cell* **32**, 185–203.e113 (2017). 28810144 10.1016/j.ccell.2017.07.007 PMC5964983

[39] Zhang, S. et al. The UBE2C/CDH1/DEPTOR axis is an oncogene and tumor suppressor cascade in lung cancer cells. *J. Clin. Invest* **133**, e162434 (2023). 36548081 10.1172/JCI162434 PMC9927933

[40] Waddell, N. et al. Whole genomes redefine the mutational landscape of pancreatic cancer. *Nature* **518**, 495–501 (2015). 25719666 10.1038/nature14169 PMC4523082

[41] Thomas, D. & Radhakrishnan, P. Tumor-stromal crosstalk in pancreatic cancer and tissue fibrosis. *Mol. Cancer* **18**, 14 (2019). 30665410 10.1186/s12943-018-0927-5 PMC6341551

[42] Raphael, B. J., Dobson, J. R., Oesper, L. & Vandin, F. Identifying driver mutations in sequenced cancer genomes: computational approaches to enable precision medicine. *Genome Med.* **6**, 5 (2014). 24479672 10.1186/gm524 PMC3978567

[43] Patel, N. M. et al. Improved tumor purity metrics in next-generation sequencing for clinical practice: the integrated interpretation of neoplastic cellularity and sequencing results (IINCaSe) approach. *Appl Immunohistochem. Mol. Morphol.* **27**, 764–772 (2019). 30102605 10.1097/PAI.0000000000000684 PMC6887630

[44] Hong, T. H. et al. Clinical advantage of targeted sequencing for unbiased tumor mutational burden estimation in samples with low tumor purity. *J. Immunother. Cancer* **8**, e001199 (2020). 33077514 10.1136/jitc-2020-001199 PMC7574938

[45] Chikhladze, S. et al. Adjuvant chemotherapy after surgery for pancreatic ductal adenocarcinoma: retrospective real-life data. *World J. Surg. Oncol.* **17**, 185 (2019). 31706323 10.1186/s12957-019-1732-3 PMC6842534

[46] Suto, H. et al. The predictors and patterns of the early recurrence of pancreatic ductal adenocarcinoma after pancreatectomy: the influence of pre- and post- operative adjuvant therapy. *BMC Surg.* **19**, 186 (2019). 31796066 10.1186/s12893-019-0644-z PMC6891951

[47] Ratnayake, B. et al. Recurrence patterns for pancreatic ductal adenocarcinoma after upfront resection versus resection following neoadjuvant therapy: a comprehensive meta-analysis. *J. Clin. Med*. **9**, 2132 (2020). 32640720 10.3390/jcm9072132 PMC7408905

