# Supplementary material for: Multiomic quantification of the KRAS mutation dosage improves the preoperative prediction of survival and recurrence in patients with pancreatic ductal adenocarcinoma
Source: Exp Mol Med. 2025 Jan 8;57(1):193–203. doi: 10.1038/s12276-024-01382-0 (PMC11799340; doi:10.1038/s12276-024-01382-0)
Supplement: Supplementary file 1 — Supplementary Information [file 12276_2024_1382_MOESM1_ESM.pdf]

Supplementary Information

Multiomic quantification of the KRAS mutation dosage improves the preoperative prediction of survival and recurrence in patients with pancreatic ductal adenocarcinoma

Won-Gun Yun, Daeun Kim, Youngmin Han, Wooil Kwon, Seong-geun Lee, Jin-Young Jang, Daechan Park

Contents

SUPPLEMENTARY FIGURES 3

Supplementary Fig. 1 3

Supplementary Fig. 2 4

Supplementary Fig. 3 5

Supplementary Fig. 4 6

Supplementary Fig. 5 7

Supplementary Fig. 6 8

Supplementary Fig. 7 9

Supplementary Fig. 8 10

Supplementary Fig. 9 11

Supplementary Fig. 10 12

Supplementary Fig. 11 13

Supplementary Fig. 12 14

Supplementary Fig. 13 15

Supplementary Fig. 14 17

SUPPLEMENTARY TABLES **18**

Supplementary Table 1 18

Supplementary Table 2 19

Supplementary Table 3 20

Supplementary Table 4 21

Supplementary Table 5 22

Supplementary Table 6 23

SUPPLEMENTARY FIGURES

Supplementary Fig. 1 Identifying cutoff values to define high and low dosage groups based on TS.

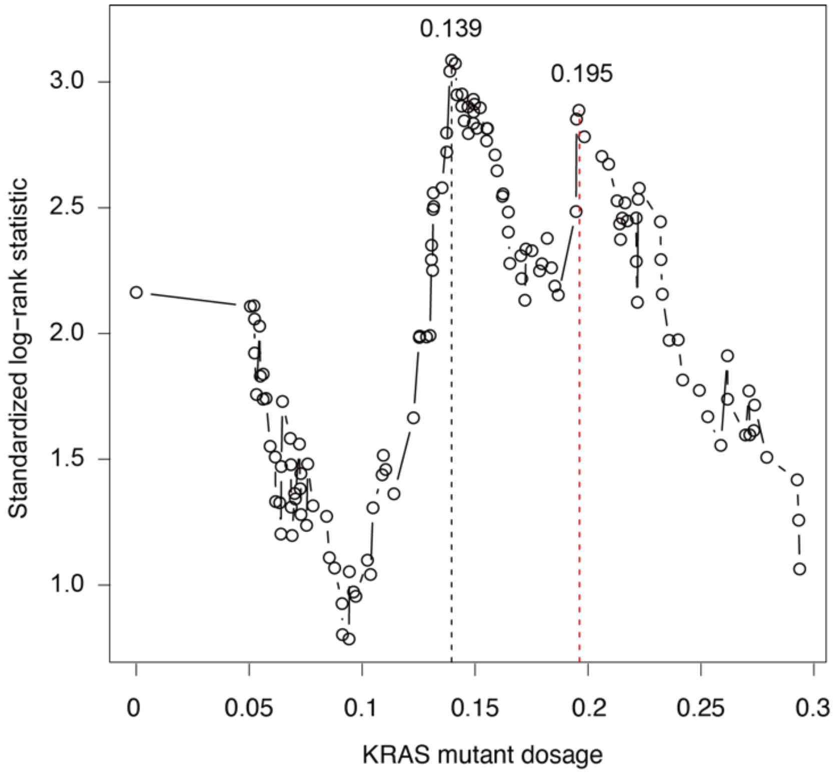

Supplementary Fig. 2 (a-f) Comparison of SMG mutant dosage between patients who experienced recurrence and those who did not. *P*-values were calculated using the Wilcoxon signed-rank test.

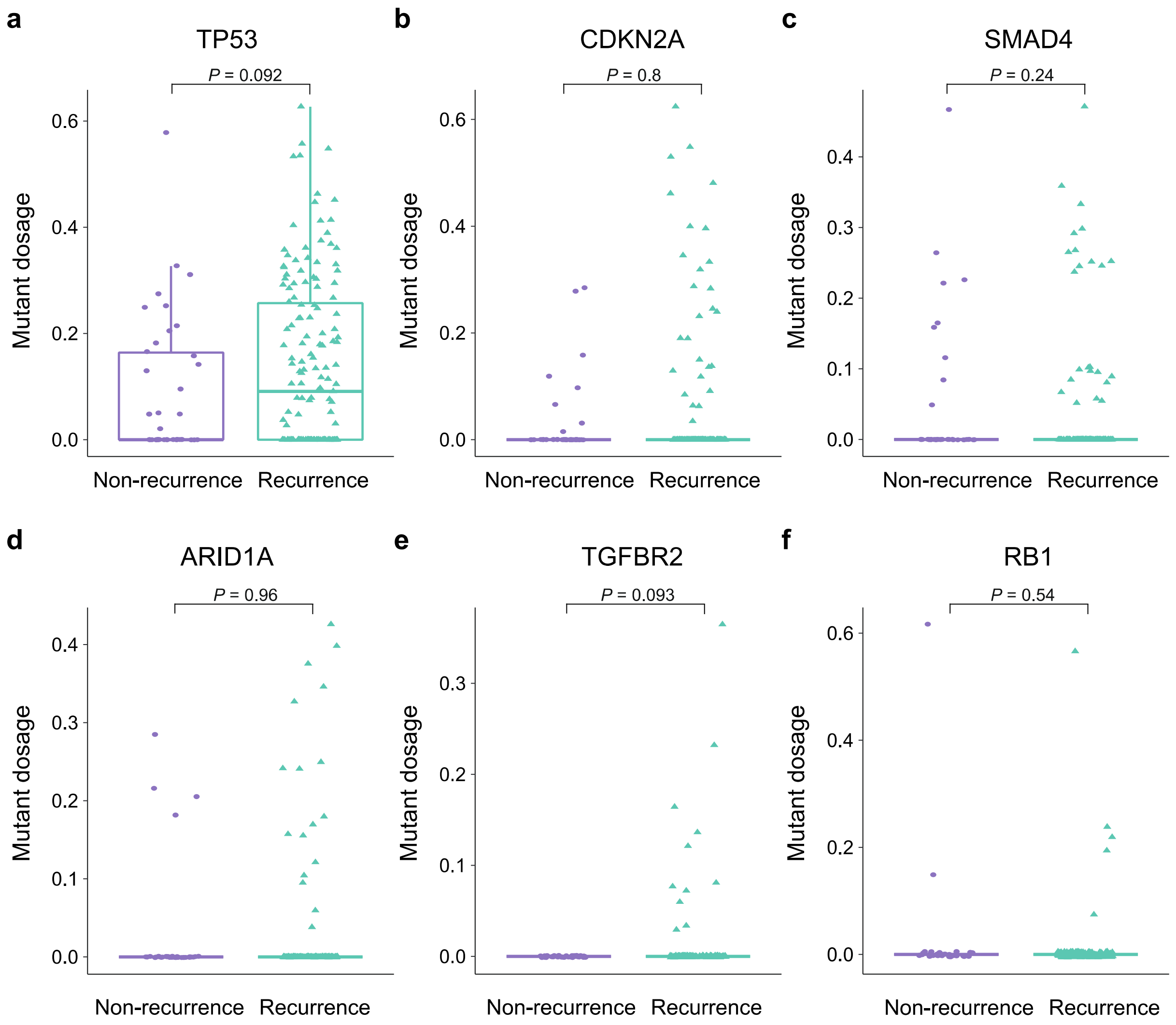

Supplementary Fig. 3 Protein–protein interaction network of differentially expressed genes (DEGs) upregulated in the low *KRAS* dosage group. The size of the circles (nodes) and the thickness of the lines (edges) are proportional to $\log_2$ fold-change (FC) and the degree of protein–protein interactions, respectively. Genes are color-coded according to their biological functions.

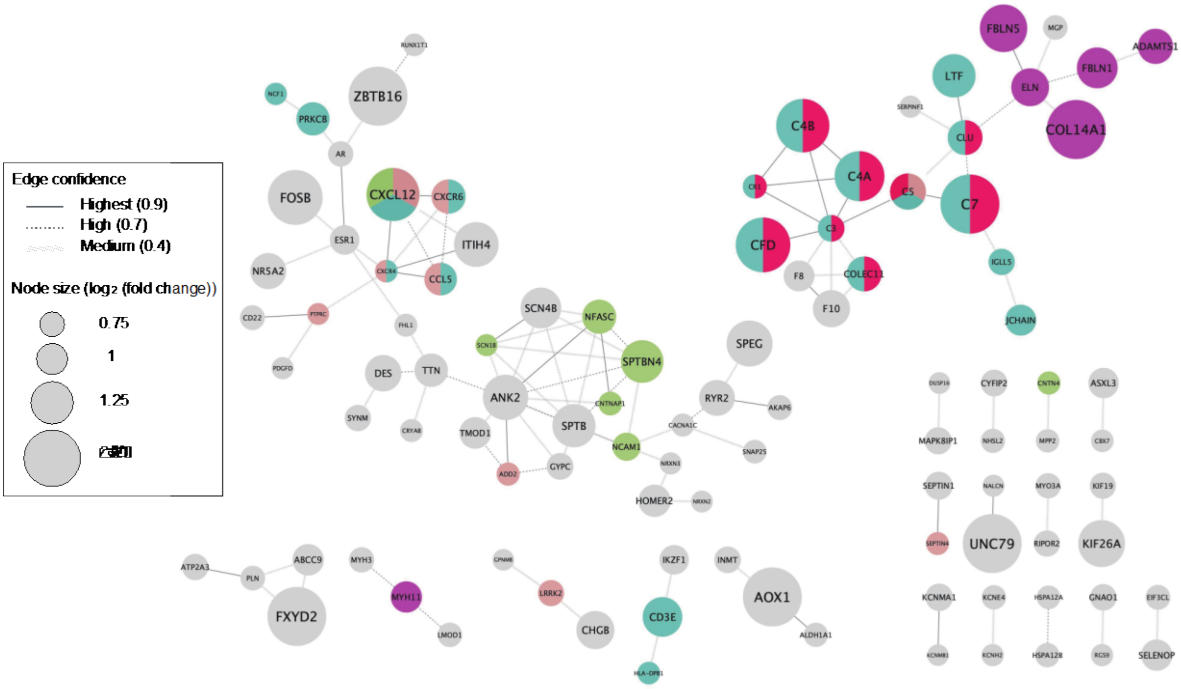

Supplementary Fig. 4 Timing of recurrence according to the prognostic scores.

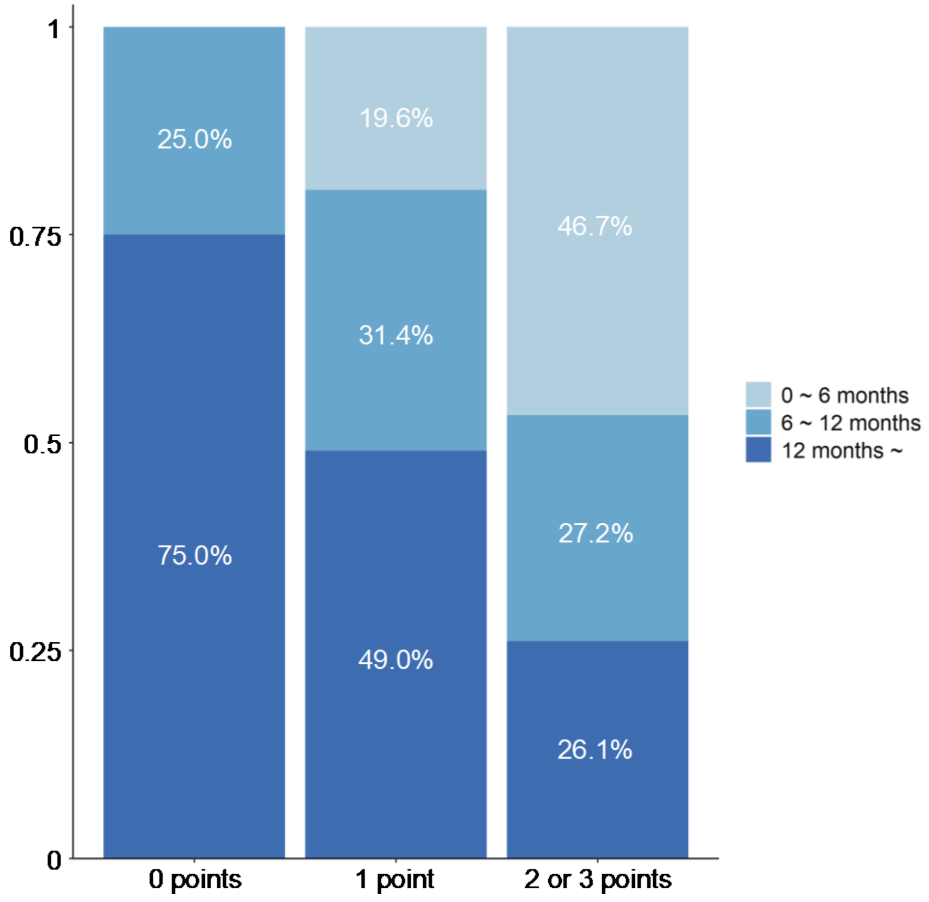

Supplementary Fig. 5 (a-b) Overall and recurrence-free survival according to prognostic scoring systems with only clinical variables, (c-d) mutant dosage from RNA sequencing, and (e-f) mutant dosage from whole exome sequencing. *, $P < 0.05$; **, $P < 0.01$; ***, $P < 0.001$ obtained using log-rank. *NS*, no statistically significant difference.

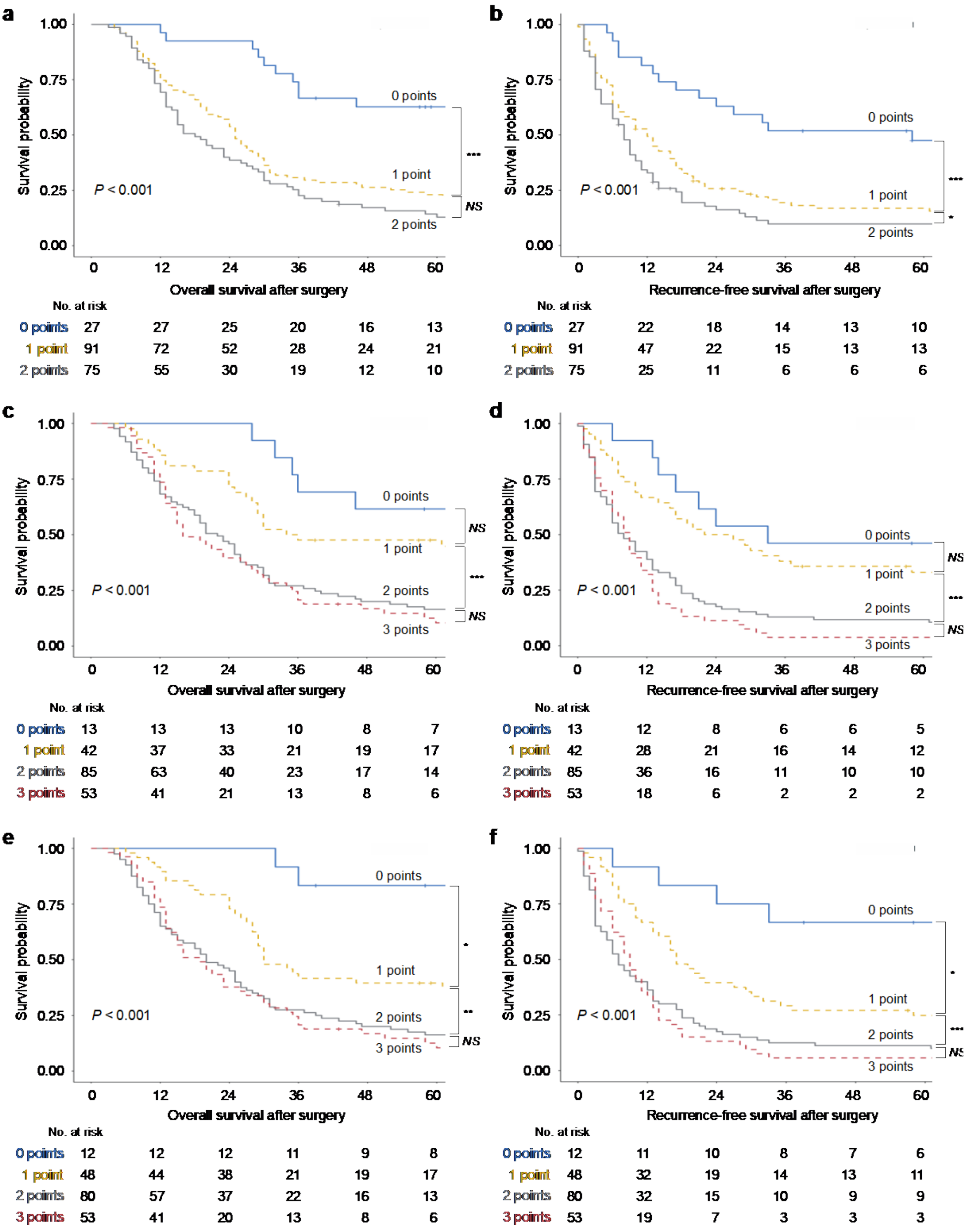

Supplementary Fig. 6 Overall survival between patients with high (> 0.139) and low (≤ 0.139) *KRAS* mutant dosages of TS. Survival analysis was performed using the Kaplan–Meier curve and log-rank test.

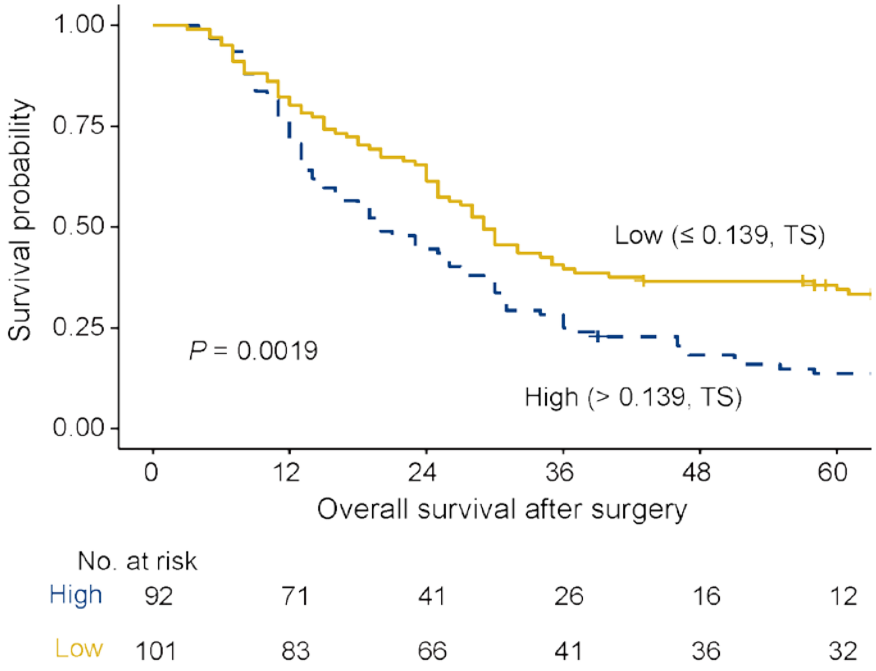

Supplementary Fig. 7 Analysis of DEGs between high (> 0.139, n = 92) and low (≤ 0.139, n = 101) *KRAS* mutant dosage groups in TS. (a) Volcano plot showing DEGs in the high *KRAS* dose group compared to the low *KRAS* dose group. DEGs satisfying the following criteria were considered significant: $-\log_{10}$ (adjusted p-value) < 0.001 and the absolute value of $\log_2$ (fold change) ≥ 0.5 (b) Heat map of 769 significant DEGs between high and low *KRAS* dosage groups. (c-d) Gene Ontology (GO) enrichment analysis of DEGs upregulated in the high or low *KRAS* dosage group.

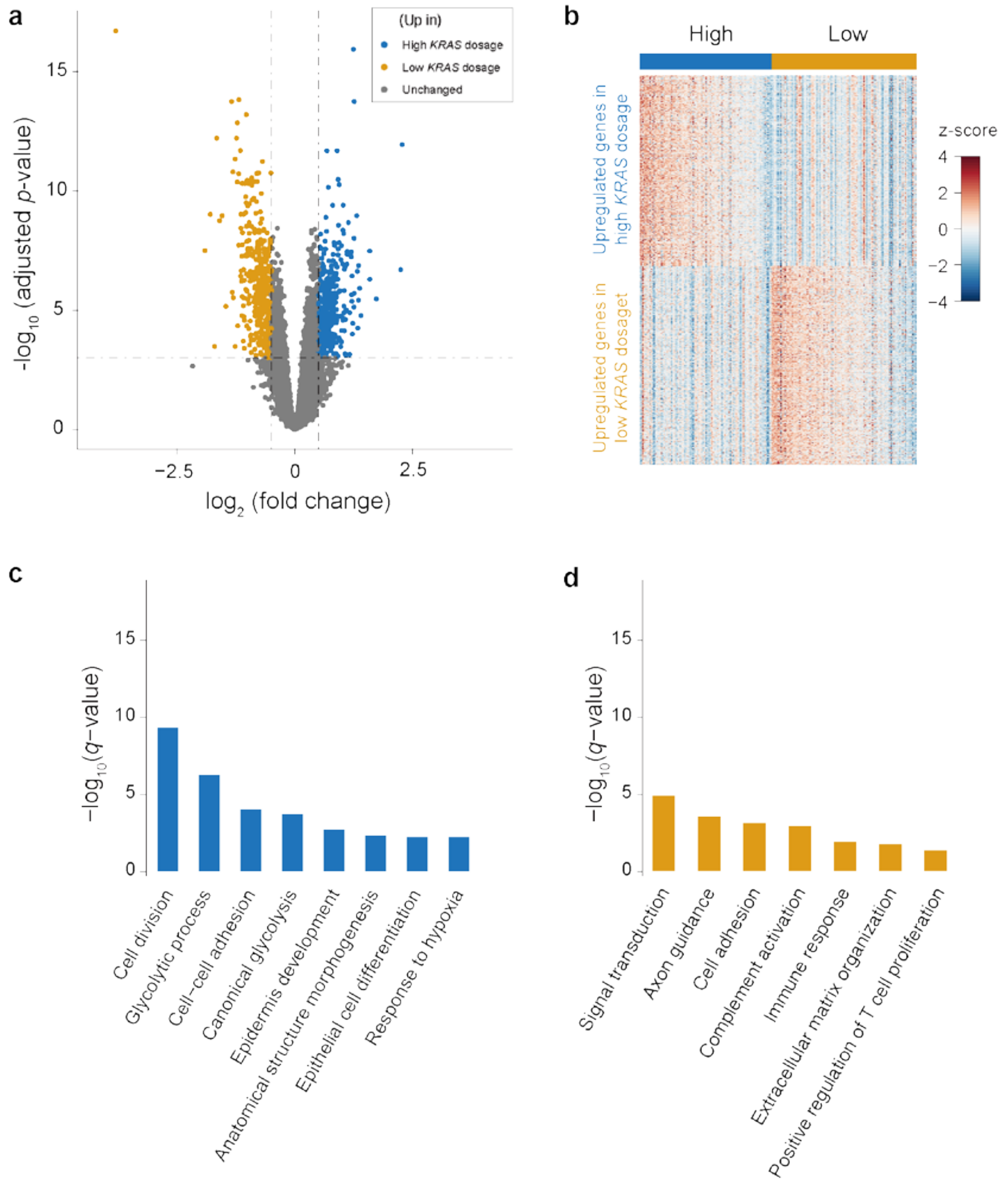

Supplementary Fig. 8 Network model describing the interactions of DEGs upregulated in the high (> 0.139) *KRAS* mutant dose group. The size of circles (nodes) and thickness of lines (edges) are proportional to log_2(fold change) and the degree of protein–protein interactions, respectively. Genes are color-coded according to their biological functions.

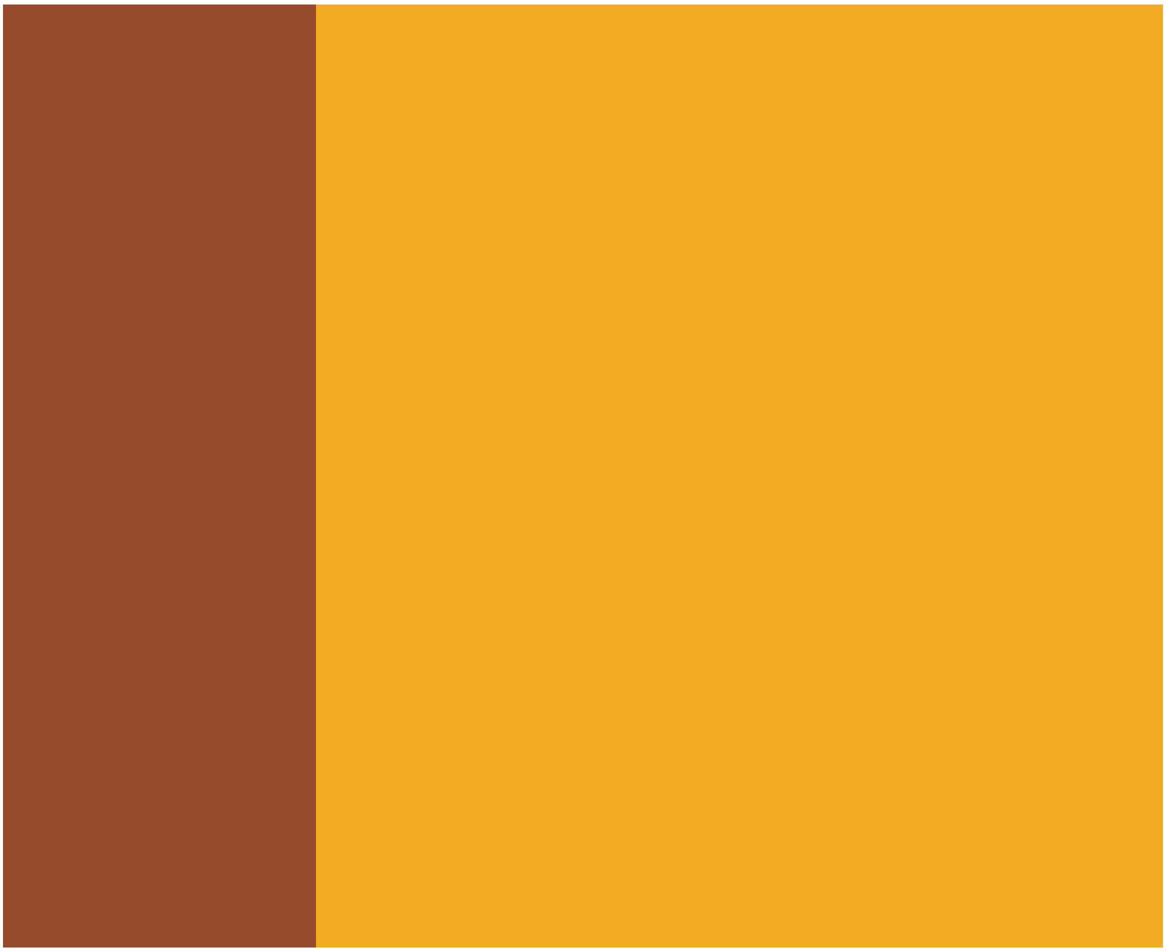

Supplementary Fig. 9 Network model describing interactions of differentially expressed genes (DEGs) upregulated in the low (≤ 0.139) *KRAS* mutant dosage group. The size of circles (nodes) and thickness of lines (edges) are proportional to $\log_2$(fold change) and the degree of protein–protein interactions, respectively. Genes are color-coded according to their biological functions.

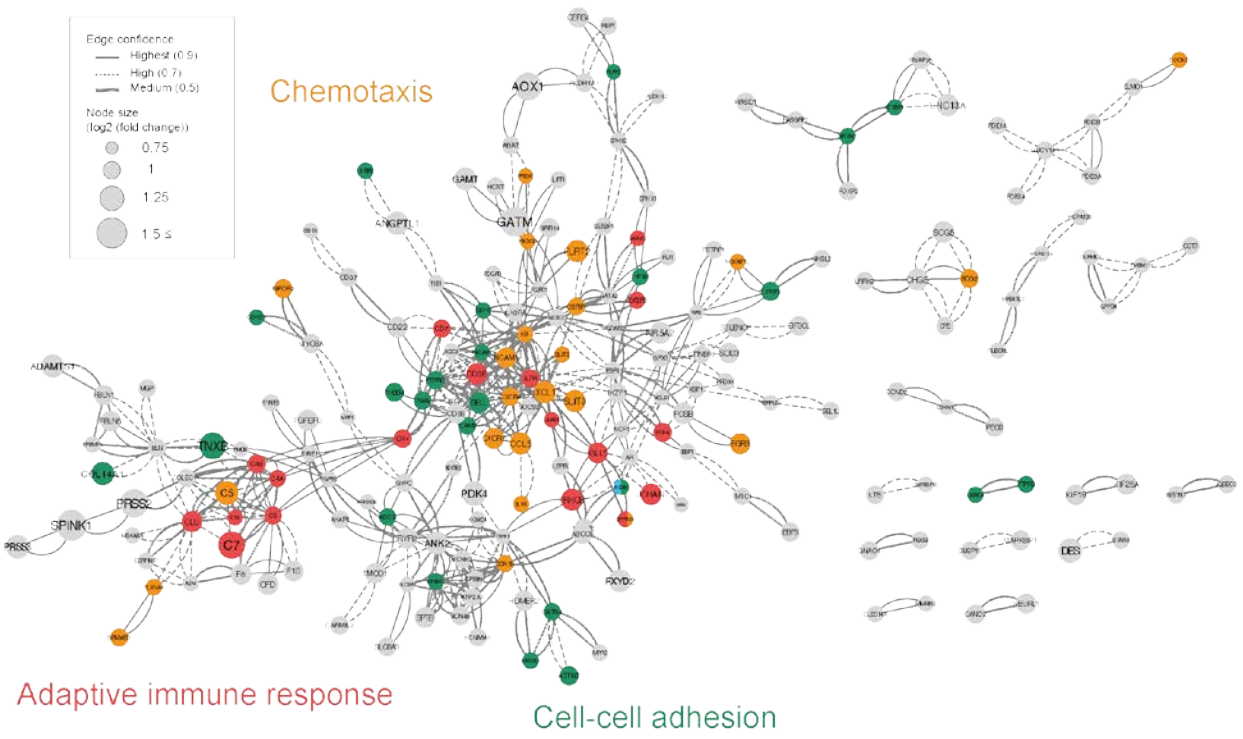

Supplementary Fig. 10 (a-b) Overall and recurrence-free survival according to prognostic scores by integrating *KRAS* G12 mutant dosage (cutoff: 0.139) from *KRAS* targeted sequencing with clinical variables. Survival analysis was performed using the Kaplan–Meier curve and log-rank test.

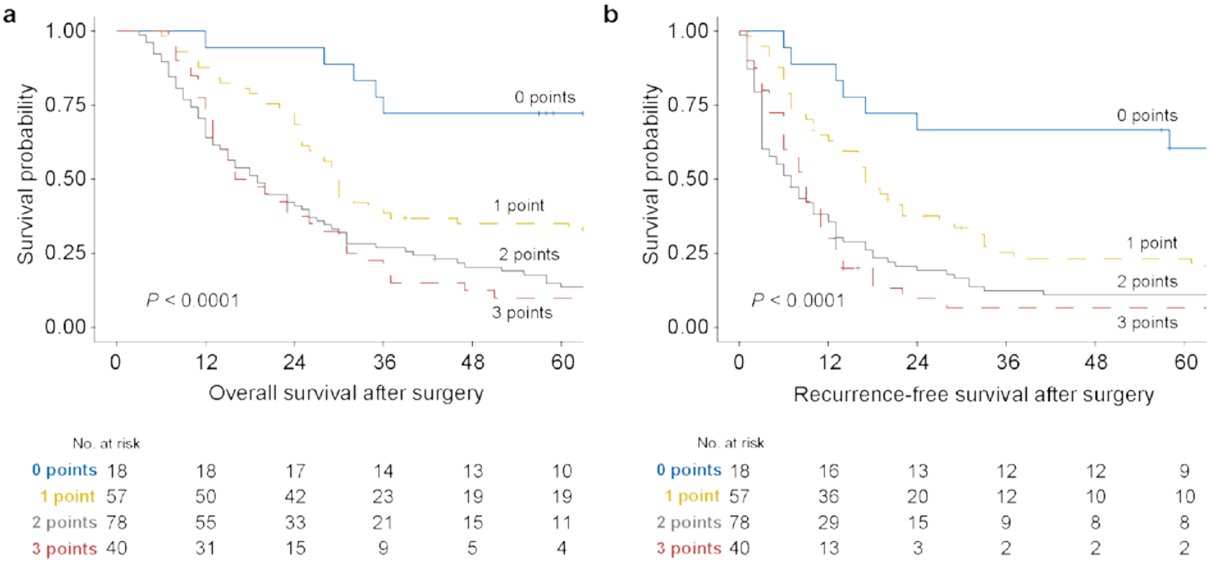

Supplementary Fig. 11 Tumor purity according to *KRAS* G12 mutant dosage from RNA sequencing (a), whole exome sequencing (b), and *KRAS* targeted sequencing (c). Tumor size according to mutant dosage based on *KRAS* targeted sequencing (d).

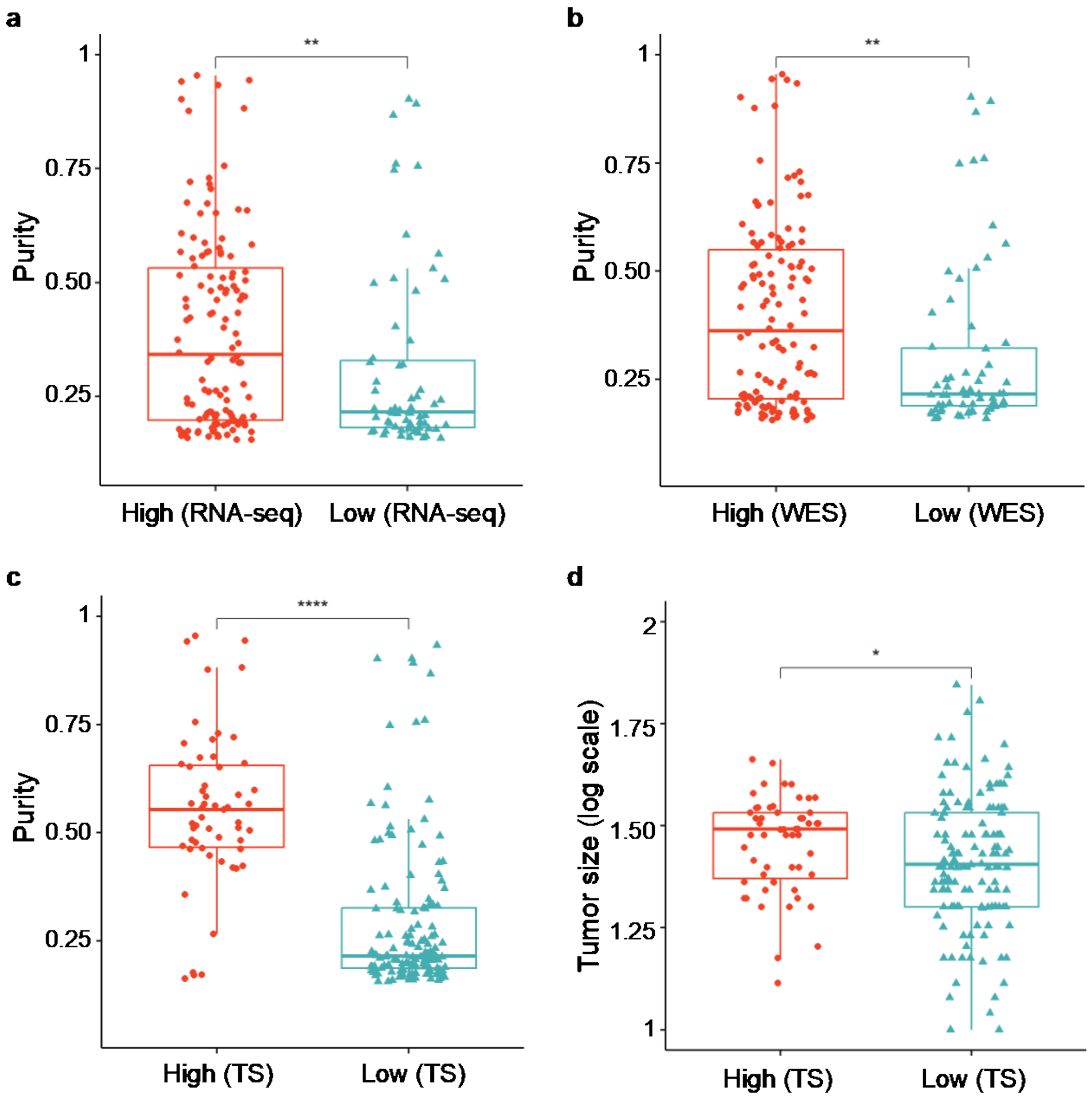

Supplementary Fig. 12 Overall survival between high and low *KRAS* mutant dosage groups from (a) RNA-seq, (b) WES, and (c) TS adjusted for copy number variations. Survival analysis was performed using the Kaplan-Meier curve and log-rank test. RNA-seq, RNA sequencing; TS, *KRAS* targeted sequencing; WES, whole exome sequencing; High, high *KRAS* mutant dosage group; Low, low *KRAS* mutant dosage group.

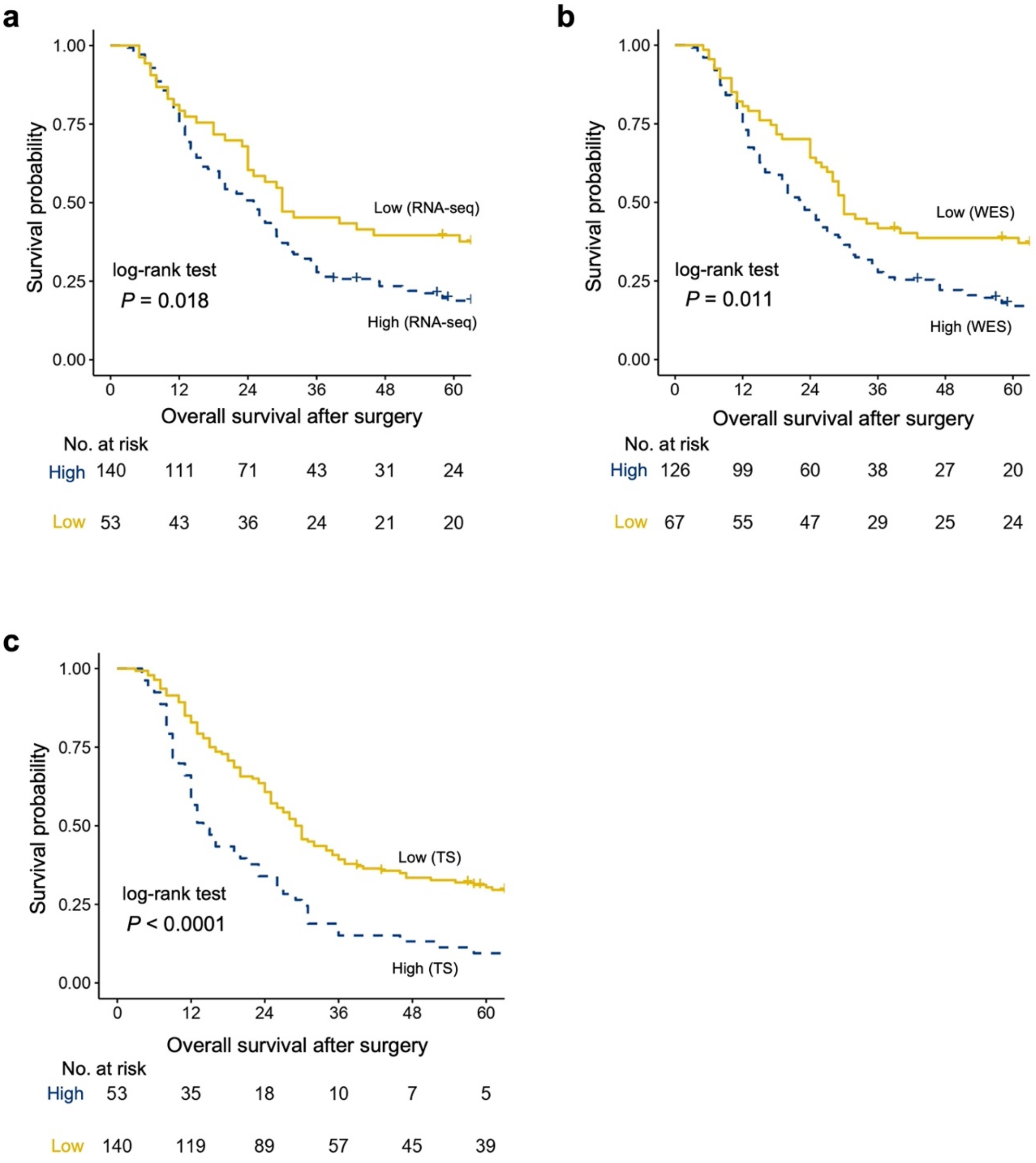

Supplementary Fig. 13 Overall and recurrence-free survival between the high and low *KRAS* mutant dosage groups with similar tumor cellularity. (a-b) Survival analysis of samples with tumor purity ranging from 0.16 to 0.36. (c-d) Survival analysis of samples with tumor purity ranging from 0.36 to 0.5 (e-f) Survival analysis of samples with high tumor purity (> 0.419, n = 71). The cutoff for stratifying samples into high and low tumor purity groups was determined using a log-rank test. (g-h) Survival analysis of samples with low tumor purity (≤ 0.419, n = 122). Survival analysis was performed using the Kaplan–Meier curve and log-rank test. High indicates high *KRAS* mutant dosage group; low indicates low *KRAS* mutant dosage group.

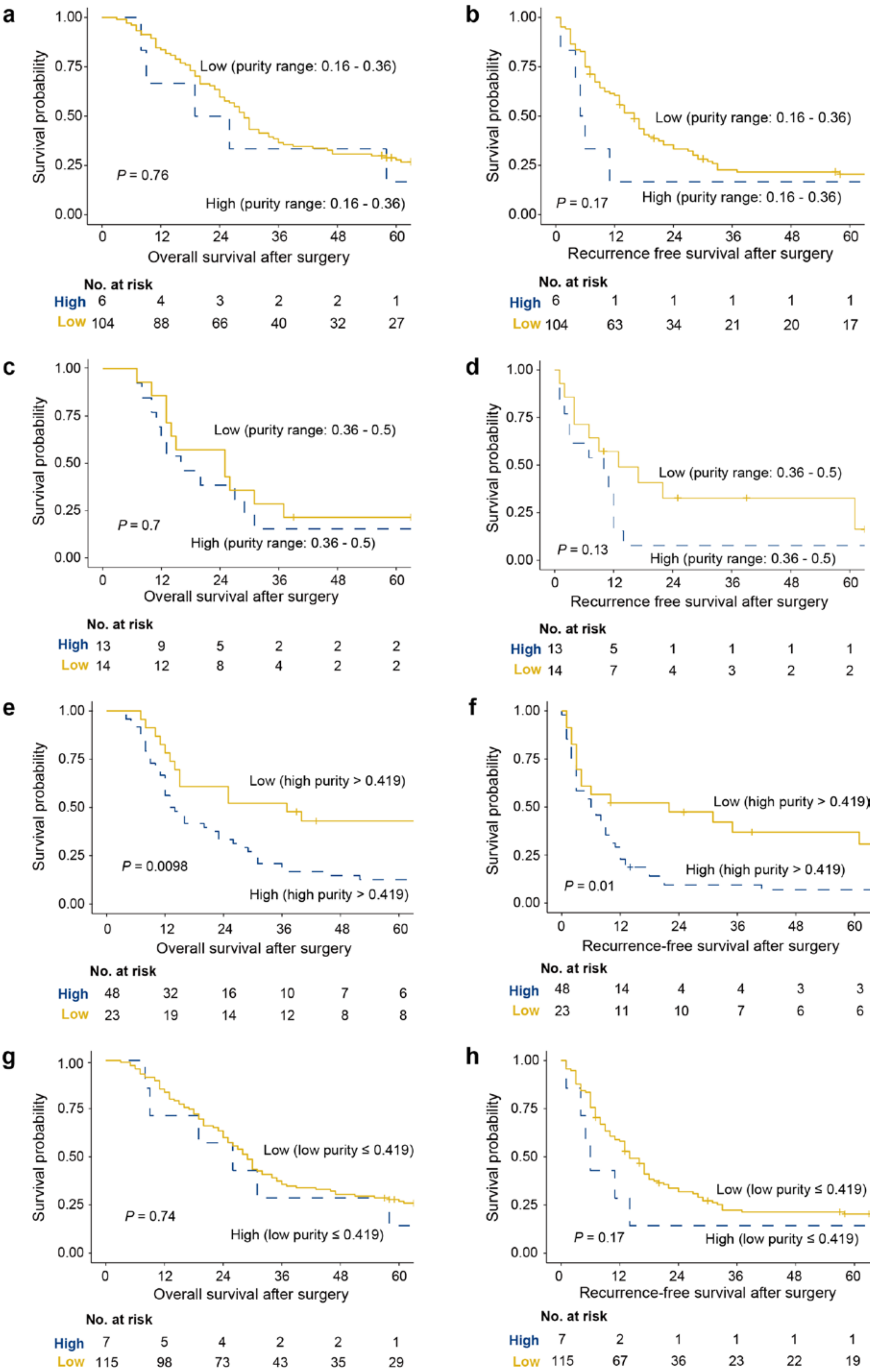
a
Survival probability
1.00
0.75
0.50
0.25
0.00
Low (purity range: 0.16 - 0.36)
P = 0.76
High (purity range: 0.16 - 0.36)
0 12 24 36 48 60
Overall survival after surgery
No. at risk
High 6 4 3 2 2 1
Low 104 88 66 40 32 27
b
Survival probability
Low (purity range: 0.16 - 0.36)
P = 0.17
High (purity range: 0.16 - 0.36)
Recurrence free survival after surgery
No. at risk
High 6 1 1 1 1 1
Low 104 63 34 21 20 17
c
Survival probability
Low (purity range: 0.36 - 0.5)
P = 0.7
High (purity range: 0.36 - 0.5)
Overall survival after surgery
No. at risk
High 13 9 5 2 2 2
Low 14 12 8 4 2 2
d
Survival probability
Low (purity range: 0.36 - 0.5)
P = 0.13
High (purity range: 0.36 - 0.5)
Recurrence free survival after surgery
No. at risk
High 13 5 1 1 1 1
Low 14 7 4 3 2 2
e
Survival probability
Low (high purity > 0.419)
P = 0.0098
High (high purity > 0.419)
Overall survival after surgery
No. at risk
High 48 32 16 10 7 6
Low 23 19 14 12 8 8
f
Survival probability
Low (high purity > 0.419)
P = 0.01
High (high purity > 0.419)
Recurrence-free survival after surgery
No. at risk
High 48 14 4 4 3 3
Low 23 11 10 7 6 6
g
Survival probability
Low (low purity ≤ 0.419)
P = 0.74
High (low purity ≤ 0.419)
Overall survival after surgery
No. at risk
High 7 5 4 2 2 1
Low 115 98 73 43 35 29
h
Survival probability
Low (low purity ≤ 0.419)
P = 0.17
High (low purity ≤ 0.419)
Recurrence-free survival after surgery
No. at risk
High 7 2 1 1 1 1
Low 115 67 36 23 22 19

Supplementary Fig. 14 (a-b) Overall and recurrence-free survival according to *KRAS* mutant dosage in TS. Patients with subclonal mutations and high Q61 variant allele frequency (> 0.195) experienced group membership transitions from low to high. Survival analysis was performed using the Kaplan–Meier curve and log-rank test.

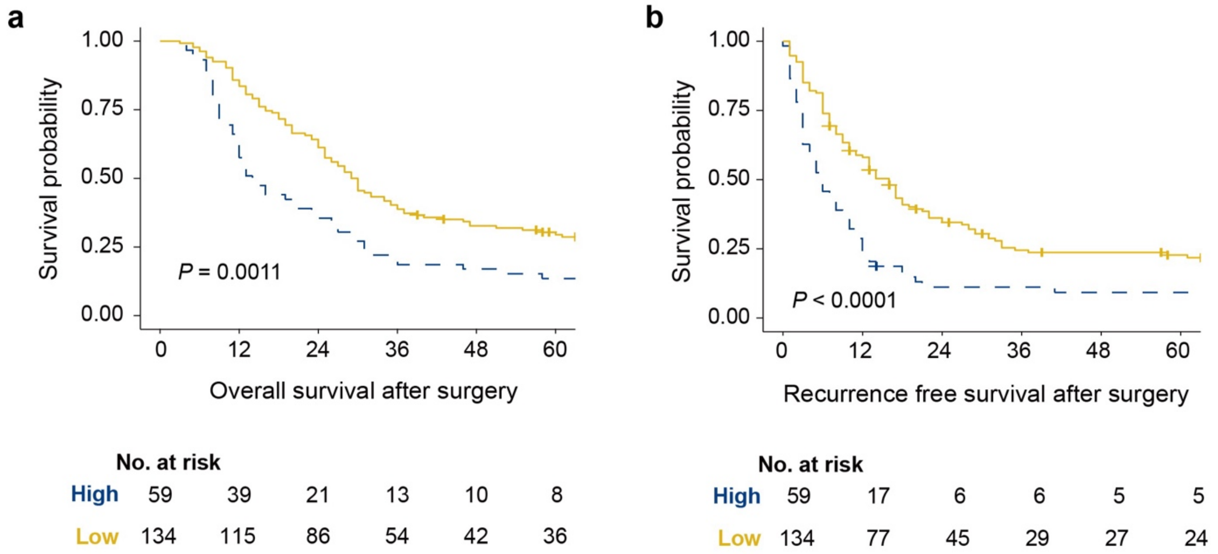

SUPPLEMENTARY TABLES

Supplementary Table 1 Mutational rate of SMGs according to the recurrence

Variables	Recurrence	Non-recurrence	*P*-value
KRAS (TS)			0.022
Wildtype	27 (17.4)	13 (34.2)	
Mutant	128 (82.6)	25 (65.8)	
KRAS (WES)			0.071
Wildtype	42 (27.1)	16 (42.1)	
Mutant	113 (72.9)	22 (57.9)	
KRAS (RNA-seq)			0.123
Wildtype	2 (1.3)	2 (5.3)	
Mutant	153 (98.7)	36 (94.7)	
TP53			0.234
Wildtype	65 (41.9)	20 (52.6)	
Mutant	90 (58.1)	18 (47.4)	
CDKN2A			0.602
Wildtype	128 (82.6)	30 (78.9)	
Mutant	27 (17.4)	8 (21.1)	
SMAD4			0.229
Wildtype	131 (84.5)	29 (76.3)	
Mutant	24 (15.5)	9 (23.7)	
ARID1A			0.938
Wildtype	138 (89.0)	34 (89.5)	
Mutant	17 (11.0)	4 (10.5)	
TGFBR2			0.091
Wildtype	144 (92.9)	38 (100.0)	
Mutant	11 (7.1)	0 (0.0)	
RB1			0.547
Wildtype	150 (96.8)	36 (94.7)	
Mutant	5 (3.2)	2 (5.3)	

Abbreviations: RNA-seq, RNA sequencing; TS, *KRAS* targeted sequencing; WES, whole exome sequencing

Supplementary Table 2 Prognostic factors among clinical variables

Variables		Overall survival		Recurrence-free survival	
		HR (95% CI)	*P*-value	HR (95% CI)	*P*-value
Age (year)	> 65	1.0 (Reference)	NA	1.0 (Reference)	NA
	≤ 65	0.87 (0.64, 1.19)	0.386	0.94 (0.70, 1.28)	0.702
Sex	Male	1.0 (Reference)	NA	1.0 (Reference)	NA
	Female	0.93 (0.68, 1.27)	0.642	0.96 (0.70, 1.30)	0.771
ASA classification	I / II	1.0 (Reference)	NA	1.0 (Reference)	NA
	III / IV	1.23 (0.71, 2.14)	0.453	1.34 (0.77, 2.33)	0.294
Location	Head	1.0 (Reference)	NA	1.0 (Reference)	NA
	Body/Tail	0.68 (0.49, 0.94)	0.018	0.76 (0.56, 1.04)	0.088
	Diffuse	0.61 (0.19, 1.92)	0.394	0.49 (0.15, 1.54)	0.221
Tumor diameter (mm)	> 20	1.0 (Reference)	NA	1.0 (Reference)	NA
	≤ 20	0.56 (0.38, 0.84)	0.005	0.55 (0.37, 0.82)	0.003
CA 19-9 (U/mL)	> 150	1.0 (Reference)	NA	1.0 (Reference)	NA
	≤ 150	0.53 (0.38, 0.72)	< 0.001	0.47 (0.35, 0.65)	< 0.001
Pathological stage	I	1.0 (Reference)	NA	1.0 (Reference)	NA
	II	1.58 (1.07, 2.33)	0.021	1.41 (0.98, 2.04)	0.065
	III	2.15 (1.37, 3.38)	< 0.001	2.53 (1.63, 3.92)	< 0.001
Resection margin	R0	1.0 (Reference)	NA	1.0 (Reference)	NA
	R1	0.85 (0.57, 1.28)	0.435	0.74 (0.50, 1.09)	0.127
Adjuvant CTx.	Y	1.0 (Reference)	NA	1.0 (Reference)	NA
	N	1.74 (1.25, 2.44)	0.001	1.84 (1.33, 2.54)	< 0.001

Abbreviations: ASA, American Society of Anesthesiologists; CA 19-9, carbohydrate antigen; CI, confidence interval; CTx., chemotherapy; HR, hazard ratio; NA, not applicable

Supplementary Table 3 Baseline clinical characteristics according to TS *KRAS* G12 mutant dosage (TS cutoff: 0.139)

Variables	Total	High (> 0.139, TS)	Low (≤ 0.139, TS)	*P*-value
N (%)	193	92 (47.7)	101 (52.3)	
Age, mean (SD), y	64.7 (10.2)	64.6 (10.6)	64.9 (9.9)	0.825
Sex				0.894
Male	109 (56.5)	51 (55.4)	58 (57.4)	
Female	84 (43.5)	41 (44.6)	43 (42.6)	
ASA classification				0.514
I / II	179 (92.7)	87 (94.6)	92 (91.1)	
III / IV	14 (7.3)	5 (5.4)	9 (8.9)	
Location				0.466
Head	109 (56.5)	48 (52.2)	61(60.4)	
Body/Tail	80 (41.5)	42 (45.6)	38 (37.6)	
Diffuse	4 (2.0)	2 (2.2)	2 (2.0)	
Tumor size, mm				0.489
> 20	150 (77.7)	74 (80.4)	76 (75.2)	
≤ 20	43 (22.3)	18 (19.6)	25 (24.8)	
CA 19-9, U/mL				0.139
> 150	91 (47.2)	49 (53.3)	42 (41.6)	
≤ 150	102 (52.8)	43 (46.7)	59 (58.4)	
Pathological stage				0.927
I	55 (28.5)	25 (27.2)	30 (29.7)	
II	97 (50.3)	47 (51.1)	50 (49.5)	
III	41 (21.2)	20 (21.7)	21 (20.8)	
Resection margin				0.216
R0	160 (82.9)	80 (87.0)	80 (79.2)	
R1	33 (17.1)	12 (13.0)	21 (20.8)	
Adjuvant CTx.				0.789
Y	135 (69.9)	63 (68.5)	72 (71.3)	
N	58 (30.1)	29 (31.5)	29 (28.7)	

Abbreviations: ASA, American Society of Anesthesiologists; CA 19-9, carbohydrate antigen 19-9; CTx., chemotherapy; RNA-seq, RNA sequencing; TS, *KRAS* targeted sequencing; WES, whole exome sequencing

Supplementary Table 4 Recurrence-related factors according to TS *KRAS* G12 mutant dosage (TS cutoff: 0.139)

Variables	High (> 0.139, TS)	Low (≤ 0.139, TS)	*P*-value
Recurrence			0.006
Y	82 (89.1)	73 (72.3)	
N	10 (10.9)	28 (27.7)	
Recurrence pattern[a]			0.563
Local	16 (19.5)	18 (24.7)	
Distant	66 (80.5)	55 (75.3)	
Recurrence timing[a]			0.293
Very early	31 (37.8)	22 (30.1)	
Early	25 (30.5)	19 (26.0)	
Late	26 (31.7)	32 (43.9)	
Recurrence timing[a]			0.211
Early (< 24 months)	75 (91.5)	61 (83.6)	
Late (≥ 24 months)	7 (8.5)	12 (16.4)	

Abbreviations: RNA-seq, RNA sequencing; TS, *KRAS* targeted sequencing; WES, whole exome sequencing

[a]Only in patients who experienced recurrence.

Supplementary Table 5 Correlation of the prognostic scoring system (TS cutoff: 0.139) with overall and recurrence-free survival outcomes

			Overall Survival			
Scores (% of patients)	HR (95% CI)	*P*-value	Median, month	1-year	3-year	5-year
0 points (9.3%)	1.00 (Reference)	NA	Not reached	94.4%	72.2%	72.2%
1 point (29.6%)	3.50 (1.49, 8.22)	0.004	30.0	87.7%	38.6%	35.0%
2 points (40.4%)	5.89 (2.55, 13.58)	< 0.001	19.0	64.1%	26.9%	13.6%
3 points (20.7%)	6.17 (2.60, 14.69)	< 0.001	17.5	72.5%	17.5%	10.0%
			Recurrence-Free Survival			
Scores (% of patients)	HR (95% CI)	*P*-value	Median, month	1-year	3-year	5-year
0 points (9.3%)	1.00 (Reference)	NA	Not reached	88.9%	66.7%	60.6%
1 point (29.6%)	2.83 (1.27, 6.31)	0.011	17.0	63.1%	25.2%	23.1%
2 points (40.4%)	5.37 (2.46, 11.74)	< 0.001	7.0	35.6%	12.4%	11.0%
3 points (20.7%)	5.81 (2.56, 13.18)	< 0.001	9.0	30.0%	6.7%	6.7%

Abbreviations: CI, confidence interval; HR, hazard ratio; NA, not applicable

Supplementary Table 6 Subclonal mutations in *KRAS* hotspots

Sample ID	*KRAS* mutant dosage group			*KRAS* mutational hotspots					Q61 VAF
	RNA-seq	WES	TS	A9	G12	G13	V59	Q61	
PDAC272	Low	Low	Low	-	Mut	-	-	Mut	0.486
PDAC063	Low	Low	Low	-	Mut	-	-	Mut	0.401
PDAC274	Low	Low	Low	-	Mut	-	-	Mut	0.270
PDAC076	Low	Low	Low	-	Mut	-	-	Mut	0.196
PDAC129	Low	Low	Low	-	Mut	-	-	Mut	0.087
PDAC266	Low	Low	Low	-	Mut	-	-	Mut	0.083
PDAC256	Low	Low	Low	-	Mut	-	-	Mut	0.036
PDAC031	Low	Low	Low	-	Mut	-	-	Mut	0.026
PDAC146	High	Low	Low	-	Mut	-	-	Mut	0.011
PDAC155	High	Low	Low	-	Mut	-	-	Mut	0.011
PDAC156	Low	Low	Low	-	Mut	-	-	Mut	0.011
PDAC203	High	Low	Low	-	Mut	-	-	Mut	0.011
PDAC275	High	Low	Low	-	Mut	-	-	Mut	0.011
PDAC194	High	Low	Low	-	Mut	-	-	Mut	0.010
PDAC269	High	Low	Low	-	Mut	-	-	Mut	0.010

Abbreviations: VAF, variant allele frequency; RNA-seq, RNA sequencing; TS, *KRAS* targeted sequencing; WES, whole exome sequencing; High, high *KRAS* mutant dosage group; Low, low *KRAS* mutant dosage group; Mut, mutated
